# The active constituent of pine needle oil, bornyl acetate, suppresses NSCLC progression by inhibiting the PI3K/AKT/ABCB1 signaling axis

**DOI:** 10.3389/fphar.2025.1653461

**Published:** 2025-09-23

**Authors:** Jieheng Wu, Jiaming Ren, Lu Wang, Zeyang Yang, Xuanyin Wang, Ziyi Tu, Xinlei Liu, Ye Wang, Yaxuan Cao, Xu Zhu, Long Li, Maoqin Lu, Ying Zhang, Jinyi Wu, Yu Cao

**Affiliations:** 1 Department of Immunology, Guizhou Medical University, Guiyang, China; 2 Tumor Immunotherapy Technology Engineering Research Center of Guizhou Medical University, Guizhou Medical University, Guiyang, China; 3 School of Public Health, the key Laboratory of Environmental Pollution Monitoring and Disease Control, Ministry of Education, Guizhou Medical University, Guiyang, China; 4 Department of Anatomy, Guizhou Medical University, Guiyang, China; 5 Guizhou Prenatal Diagnsis Center, The Affiliated Hospital of Guizhou Medical University, Guiyang, China; 6 Department of Nursing, Guiyang Healthcare Vocational University, Guiyang, China; 7 School of Clinical Medicine, Guizhou Medical University, Guiyang, China; 8 Department of Thoracic Surgery, The Affiliated Hospital of Guizhou Medical University, Guiyang, China

**Keywords:** Bornyl acetate, NSCLC, PI3K/AKT, ABCB1, tumor progression

## Abstract

**Introduction:**

Non-small cell lung cancer (NSCLC) is a leading cause of cancer-related mortality worldwide. Although targeted therapies and immunotherapies have improved treatment outcomes, the development of drug resistance continues to limit clinical efficacy. This study aims to explore the potential therapeutic effects and molecular mechanisms of pine needle oil and its key bioactive component, bornyl acetate (BA), in NSCLC.

**Methods:**

The anti-cancer activity of BA was evaluated in vitro using two NSCLC cell lines, A549 and NCI-H460. Cell proliferation, invasion, migration, colony formation, and apoptosis were assessed. Mechanistic studies focused on the PI3K/AKT signaling pathway and ABCB1 expression levels. The AKT agonist SC79 was used to rescue phenotypic effects. In vivo, subcutaneous xenograft models generated with NCI-H460 and A549 cells were employed to examine the antitumor efficacy of BA.

**Results:**

BA significantly inhibited proliferation, invasion, migration, and colony formation in A549 and NCI-H460 cells and promoted apoptosis. Mechanistically, BA suppressed the PI3K/AKT pathway, leading to downregulation of ABCB1. The AKT activator SC79 partially reversed BA-induced inhibition of invasion and migration. In vivo, BA treatment markedly attenuated the growth of both A549 and NCI-H460 xenograft tumors.

**Discussion:**

These results demonstrate that BA exerts potent anti-tumor effects in NSCLC by inhibiting the PI3K/AKT/ABCB1 axis. The findings provide a mechanistic rationale for the development of BA and other natural product-based therapies, particularly for the treatment of drug-resistant NSCLC.

## 1 Introduction

Non-small cell lung cancer (NSCLC) accounts for approximately 85% of all lung cancer cases and is one of the leading causes of cancer-related death worldwide (Siegel et al., 2025). Although the progression of targeted therapies (e.g., epidermal growth factor receptor tyrosine kinase inhibitors, EGFR-TKIs; anaplastic lymphoma kinase inhibitors, ALK inhibitors) and immunotherapies (e.g., programmed death-1/programmed death-ligand 1 inhibitors, PD-1/PD-L1 inhibitors) has improved survival outcomes for patients, the development of resistance has severely impaired clinical efficacy (Ramalingam et al., 2020; Golding et al., 2018) (Lahiri et al., 2023; Xia et al., 2019; Liu et al., 2022a). For example, MET amplification, a key resistance mechanism in EGFR mutant NSCLC, often results in patients not responding (Lu et al., 2021; Remon et al., 2023; Wang et al., 2019) to standard therapy, and even in cases with high PD-L1 expression, the benefit of chemotherapy or immunotherapy combination regimens is limited. Resistance mechanisms involve genetic mutations (e.g., MET exon 14 skipping mutations), epigenetic alterations, and tumor microenvironment adaptation changes (Bukowski et al., 2020), and new strategies are urgently needed to overcome these barriers.

Among these mechanisms, the overexpression of ATP-binding cassette (ABC) transporters, particularly ABCB1 (also known as Multidrug Resistance Protein 1 (MDR1) or P-glycoprotein (P-gp)), is a major contributor to multidrug resistance (MDR) in NSCLC. ABCB1 effluxes a wide range of chemotherapeutic agents (e.g., paclitaxel, docetaxel, vinorelbine), leading to treatment failure. Critically, high ABCB1 expression is frequently observed in NSCLC tumors and is clinically associated with poor response to chemotherapy and reduced overall survival (Liu et al., 2022b). Therefore, targeting ABCB1 represents a promising strategy to reverse MDR and improve therapeutic outcomes.

The expression and function of ABCB1 are tightly regulated by oncogenic signaling pathways. Notably, the hyperactivated PI3K/AKT pathway, a common feature in NSCLC, has been mechanistically linked to ABCB1 upregulation. Activation of PI3K/AKT signaling can promote the nuclear translocation of transcription factors such as NF-κB, which subsequently binds to the ABCB1 promoter to enhance its transcription, thereby directly establishing a molecular axis driving chemoresistance (Sun et al., 2019; Huang et al., 2020).

Natural products have always been an important source of anticancer drugs (Naeem et al., 2022). For example, paclitaxel (derived from Taxus) (Pi et al., 2022; Wang et al., 2022) and vinorelbine (derived from Catharanthus) (Ohe et al., 2007; Zhong et al., 2021) are cornerstone drugs for NSCLC chemotherapy. Preclinical studies have further demonstrated that compounds such as curcumin (Liu et al., 2023; Tang et al., 2022) and epigallocatechin gallate (EGCG) (Zheng et al., 2024; Wang et al., 2024) have potential in modulating apoptosis and reversing drug resistance. Notably, beyond polyphenols like curcumin and EGCG, terpenoids—a vast class of plant-derived natural products that includes monoterpenes such as borneol acetate—have also demonstrated potent activity in overcoming ABCB1-mediated resistance. Several terpenoids have been shown to inhibit ABCB1 function and synergize with conventional chemotherapy, highlighting their potential as resistance-reversing agents (Laiolo et al., 2024; Fang et al., 2018). These components often have multi-target effects, making them ideal candidates for tackling complex resistance pathways. However, monoterpenes from plant sources, such as borneol acetate, have not been sufficiently studied in NSCLC.

Pine needle oil is a traditional medicinal extract containing bioactive components such as α-pinene, β-pinene and borneol acetate, and has anti-inflammatory, antioxidant and antibacterial properties (Ankney et al., 2022; Clark et al., 2014). Recent studies have revealed broader anticancer effects: pine needle oil exhibits antiproliferative and proapoptotic activity in liver cancer and breast cancer models by regulating cell cycle progression and inhibiting angiogenesis (Chen et al., 2015) (Ren et al., 2018). Notably, borneol acetate inhibits the PI3K/AKT and MAPK/ERK pathways, which are often abnormal in drug-resistant cancers. However, there have been no studies exploring the role of pine needle oil or its constituents, such as borneol acetate, in NSCLC.

In this study, we found that bornyl acetate suppresses NSCLC progression *in vitro* and *in vivo* through PI3K/AKT/ABCB1 inhibition. These findings provide preclinical evidence for natural product-based combination strategies, offering potential synergistic efficacy with reduced toxicity profiles. By addressing this research gap, we advance plant-derived adjuvant development for drug-resistant NSCLC.

## 2 Materials and methods

### 2.1 Chemicals and compounds

The following compounds were used: Pine needle oil (PNO) (MACKLIN, P905670), α-Pinene (P854547), β-Phellandrene (P816047), β-Caryophyllene (C832338), D-Limonene (D887405), Bornyl acetate (BA) (B909156), Camphene (C804854), and α-Terpinene (T819532). All compounds were obtained from MACKLIN.

### 2.2 Cell lines

Human NSCLC cell lines A549 and NCI-H460 were acquired from the American Type Culture Collection (ATCC, Manassas, VA). Cell line authentication was performed using short tandem repeat Short Tandem Repeat (STR profiling), with genetic lineages matching ATCC reference databases. *Mycoplasma* contamination was routinely excluded using PCR-based detection.

A549 cells were maintained in DMEM/F12 (Gibco, 11330032), while NCI-H460 cells were cultured in RPMI-1640 (Gibco, 11875093). Both cell media (CM) were supplemented with 10% FBS (Gibco, 10270106) and 1% penicillin-streptomycin (Gibco, 15140122). Cells were incubated at 37 °C under 5% CO_2_ and subcultured at 80%–90% confluency using 0.25% trypsin-EDTA (Gibco, 25200056).

### 2.3 Cell viability assay

Cell viability was assessed using a Cell Counting Kit-8 (CCK-8) (ApexBio, K1018). Cells (1 × 10^4^/well) were seeded in 96-well plates (n = 5 replicates/group) and allowed to adhere for 24 h. Following 48-h treatments with specified compounds, CCK-8 reagent was added and incubated for 1 h. Absorbance was measured at 450 nm using a SpectraMax microplate reader (Molecular Devices). Three independent experiments were performed. In Cell Viability Assay experiments, an ethanol vehicle control group was included. The concentration of ethanol was matched to the highest concentration present in the BA or PNO treatment groups.

### 2.4 Transwell assay

Cell migration and invasion were evaluated using Transwell chamber assays (LABSELECT, China) with polycarbonate membranes (8-μm pore size), as previously described with minor modifications.

For the migration assay, briefly, A459 and NCI-H460 cells were harvested after trypsinization, washed with phosphate-buffered saline (PBS), and resuspended in serum-free medium. Subsequently, 1 × 10^5^ cells in 200 µL of serum-free medium were seeded into the upper chamber of a 24-well Transwell insert. The lower chamber was filled with 500 µL of complete culture medium containing 10% FBS as a chemoattractant.

For the invasion assay, the upper chamber membranes were pre-coated with 50 µL of Matrigel (BD Biosciences, San Jose, CA, United States) diluted in serum-free medium (1:8 ratio) and allowed to polymerize for 4‒6 h at 37 °C. The same cells seeding procedure as for the migration assay was then followed.

After incubation for 24 h at 37 °C in a 5% CO_2_ atmosphere, non-migratory or non-invasive cells on the upper surface of the membrane were carefully removed using a cotton swab. Cells that had migrated or invaded to the lower surface were fixed with 4% paraformaldehyde for 20 min, stained with 0.1% crystal violet for 15 min, and then rinsed gently with PBS. Five randomly selected fields per chamber were imaged under an inverted microscope, and cells were counted using ImageJ software. Each experimental group was set up in triplicate, and the assay was repeated three times independently. An ethanol vehicle control group was included, with ethanol concentrations matching the highest levels used in the BA or PNO treatment groups.

### 2.5 Wound healing assay

Cell migration was assessed using a wound healing assay. Cells in the logarithmic growth phase were seeded into 6-well plates at a density of 3 × 10^5^ per well and cultured until they reached 90%–100% confluence. A uniform scratch was created in the center of each well using a sterile 200 μL pipette tip, perpendicular to the plate surface. The wells were gently rinsed three times with PBS to remove dislodged cells. Serum-free medium containing the specified concentrations of compounds was added, and the plates were returned to the incubator. Images of the same field were captured at 0, 24, and 48 h under an inverted microscope. The scratch width was measured using ImageJ software, and cell migration was calculated as follows: Migration rate = (0 h scratch width–scratch width at time point)/(0 h scratch width) × 100%. The experiment was independently repeated three times. An ethanol vehicle control was included in all assays, with ethanol concentrations consistent with the highest levels present in the BA or PNO treatment groups.

### 2.6 Western blot

The employed primary antibodies are as follows: anti-AKT (CST, #9272), anti-Phospho-Akt (Ser473) (CST, #4060), anti-PI3 Kinase p85 (CST, #4257), anti-Phospho-PI3Kinasep85 (Tyr458)/p55 (Tyr199) (CST, #4228), anti-ABCB1(CST, #13978), anti-caspase-3(CST, #14220) and anti-GADPH (Servicebio, #GB15004). Cell lysates containing target proteins were subjected to electrophoretic separation in sodium dodecyl sulfate polyacrylamide gel electrophoresis (SDS-PAGE). The separated protein was transferred from gel to PVDF membrane. After transfer, the membrane was blocked with protein-free flash blocking solution (Servicebio, #G2052) for 25 min at room temperature. Then they were treated with either of the above primary antibodies and the corresponding horseradish peroxidase (HRP) labeled secondary antibody (Servicebio, #GB23303). In Western blot experiments, the negative control (NC) group served as the ethanol vehicle control group. The ethanol concentration in this group was consistent with the highest ethanol concentration present in the BA or PNO treatment groups.

### 2.7 Colony formation assay

A549 and NCI-H460 cells were seeded in 6-well plates at a density of 1,000/well. After 24-h adherence, medium was replaced with 0.1% FBS-containing medium for synchronization. Following 48-h treatments with bornyl acetate or pine needle oil, cells were cultured for 10–14 days (media refreshed every 72 h). Colonies were fixed with 4% PFA (Servicebio, G1101), stained with 0.1% crystal violet (Solarbio, G1063), and quantified using ImageJ. Triplicate experiments were conducted. In Colony formation assay experiments, an ethanol vehicle control group was included. The concentration of ethanol was matched to the highest concentration present in the BA or PNO treatment groups.

### 2.8 RNA sequencing and GO and KEGG pathway assessment

Total RNA was extracted from A549 cells (control and BA-treated) using TRIzol reagent. RNA integrity was verified (RIN >8.0, Agilent Bioanalyzer 2,100). Libraries were prepared with poly(A) selection and sequenced (Illumina NovaSeq 6,000; 150-bp paired-end) by PTM Bio (Hangzhou). Differentially expressed genes (|log_2_FC| > 1, FDR < 0.05) were analyzed via GO enrichment (Gene Ontology Consortium) and KEGG pathway mapping (KEGG PATHWAY Database).

### 2.9 Mouse tumour experiments

Animal studies were conducted and reported in accordance with the ARRIVE guidelines. All animal experiments were approved by the Institutional Animal Care and Use Committee of Guizhou Medical University (Protocol No.2400397) and performed in compliance with international standards for laboratory animal welfare. For *in vivo* injections, BA and PNO were first dissolved in a small volume of absolute ethanol. This solution was then emulsified by brief sonication in saline containing ethanol to form a homogeneous suspension. The final concentration of ethanol in the injectate was 0.04%. The vehicle control for *in vivo* experiments consisted of the same formulation (saline with 0.04% ethanol) without BA or PNO. 2 × 10^6^ NCI-H460-luciferase cells/200 µL or 2 × 10^6^ A549 cells/200 µL media were subcutaneously introduced to nude mice. After tumor formation, mice were injected intravenously with Bornyl acetate and Pine needle oil. We carefully monitored tumor progression with bioluminescence imaging (BLI) and recorded tumor fluorescence intensity. The tumor volume was calculated as follows: V (mm^3^) = a × b^2^/2, where a and b represent the long and short diameters, respectively. Post-experiment, all procedures in mice used isoflurane (induction: 3%–5%, maintenance: 1%–3%), with doses validated for murine physiology, and then tumors were excised for imaging. Tumor tissues were processed into paraffin sections to perform: (i) Terminal deoxynucleotidyl transferase–mediated dUTP nick end labeling (TUNEL) for apoptosis assessment, (ii) Immunofluorescence (IF) staining of Ki-67 to evaluate cellular proliferation, and (iii) Hematoxylin and eosin (H&E) histopathological analysis. Major organs (heart, liver, spleen, lungs, and kidneys) were similarly sectioned for H&E staining. Serum was collected to assess hepatotoxicity by measuring alanine transaminase (ALT) and aspartate transaminase (AST) levels, and nephrotoxicity via blood urea nitrogen (BUN) and serum creatinine (Cr) quantification.

### 2.10 Statistical analyses

All data analyses were conducted using Excel 2016. Before statistical evaluation, normality and homogeneity of variance were assessed. Results are presented as mean ± SD or mean ± SEM, and comparisons between groups were made using two-tailed t-tests. A p-value of less than 0.05 was considered statistically significant.

## 3 Results

### 3.1 Bornyl acetate constitutes a component of pine needle oil

**FIGURE 1:**
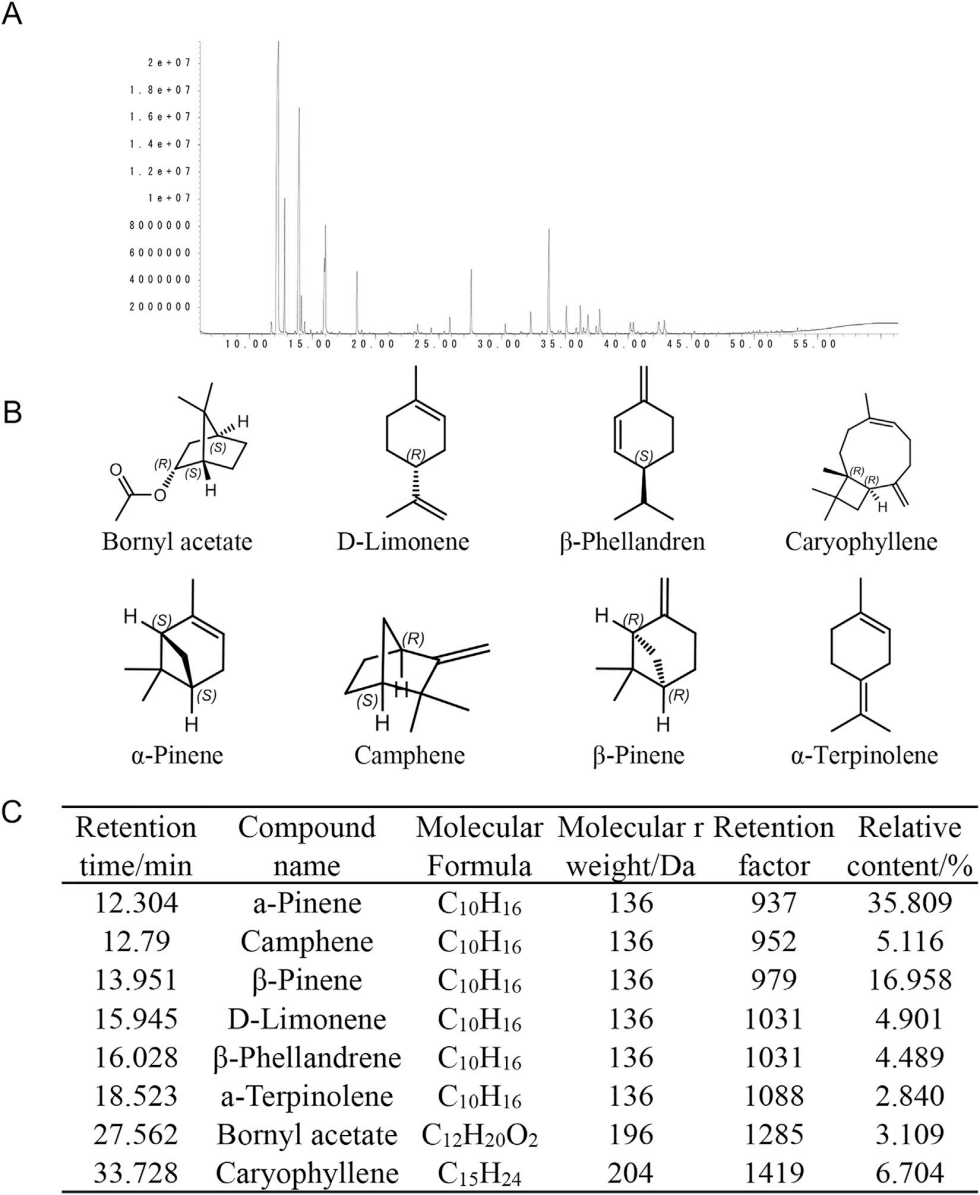
Analysis of pine needle oil and its eight active components. **(A)** Gas chromatogram of pine needle oil showing component separation. Distinct peaks represent individual chemical constituents. Abscissa: retention time (min); Ordinate: response intensity. **(B)** Chemical structures of the eight identified active components: Bornyl acetate, D-Limonene, β-Phellandrene, Caryophyllene, α-Pinene, Camphene, β-Pinene, α-Terpinene. **(C)** Analytical parameters of active components, including retention time, compound name, molecular formula, molecular weight, retention factor, and relative content.

To comprehensively characterize PNO, comprehensive compositional profiling of PNOwas performed via silica gel chromatography coupled with structural characterization. The total ion chromatogram (TIC) revealed distinct separation profiles of individual constituents (Figure 1A). Structural elucidation identified key compounds including D-limonene, BA, β-phellandrene, β-caryophyllene, α-pinene, camphene, β-pinene, and α-terpinolene (Figure 1B). Quantitative analysis demonstrated BA as a definable and consistent constituent, comprising 3.1% of total PNO (retention time: 27.562 min; molecular formula: C_12_H_20_O_2_; Figure 1C).

### 3.2 Bornyl acetate exhibits potent cytotoxicity against NSCLC cells

**FIGURE 2:**
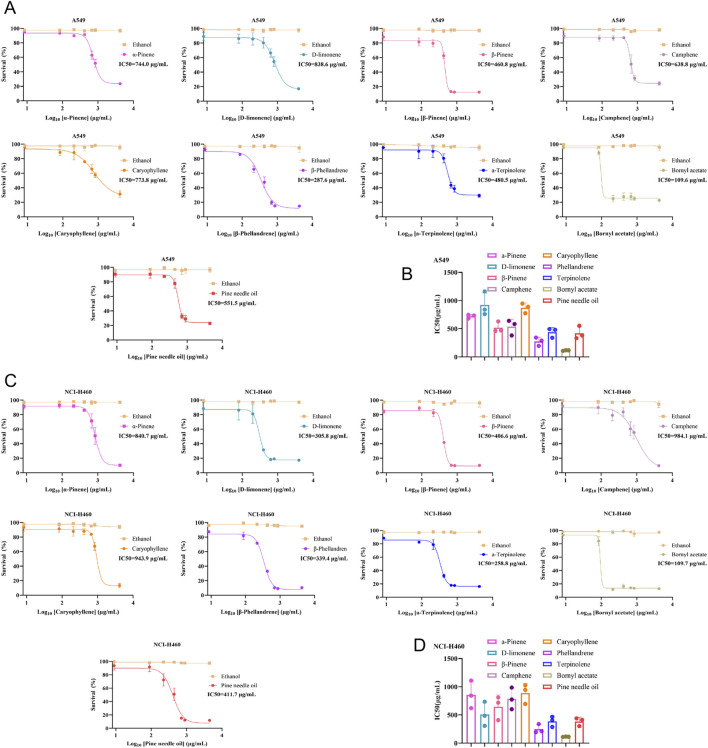
Inhibitory effects of pine needle oil and its eight active components on NSCLC cell proliferation. **(A,B)** Cell viability assessment and IC50 calculation for cisplatin-treated A549 cells cultured under monotherapy conditions (−) or co-treated with varying concentrations of Bornyl acetate, D-Limonene, β-Phellandrene, Caryophyllene, α-Pinene, Camphene, β-Pinene, α-Terpinene, or ethanol vehicle. Data represent mean ± SD (n = 3). **(C,D)** Cell viability assessment and IC50 calculation for cisplatin-treated NCI-H460 cells under identical treatment conditions. Data represent mean ± SD (n = 3).

The cytotoxic effects of PNO and eight of its active components on human NSCLC cells (A549 and NCI-H460) were evaluated using CCK-8 assays. At equivalent concentrations, PNO and its components differentially inhibited A549 cell viability, with bornyl acetate (BA) showing the most pronounced effect and the lowest Half-Maximal Inhibitory Concentration (IC_50_) values (A549: 106.9 μg/mL; *P* < 0.0001 vs. other compounds) (Figures 2A,B). Similarly, BA exhibited the strongest suppression of NCI-H460 cell growth and the lowest IC_50_ values (NCI-H460: 109.7 μg/mL; *P < 0.0001* vs. other compounds) (Figures 2C,D). Dose-response curves and comparative histograms confirm that BA possesses optimal anti-proliferative activity against NSCLC cells among PNO constituents. The ethanol vehicle, at this concentration, exhibited no significant inhibitory effect on the proliferation of either A549 or NCI-H460 cells.

### 3.3 Bornyl acetate suppresses invasion, migration, and clonogenicity in NSCLC cells

**FIGURE 3:**
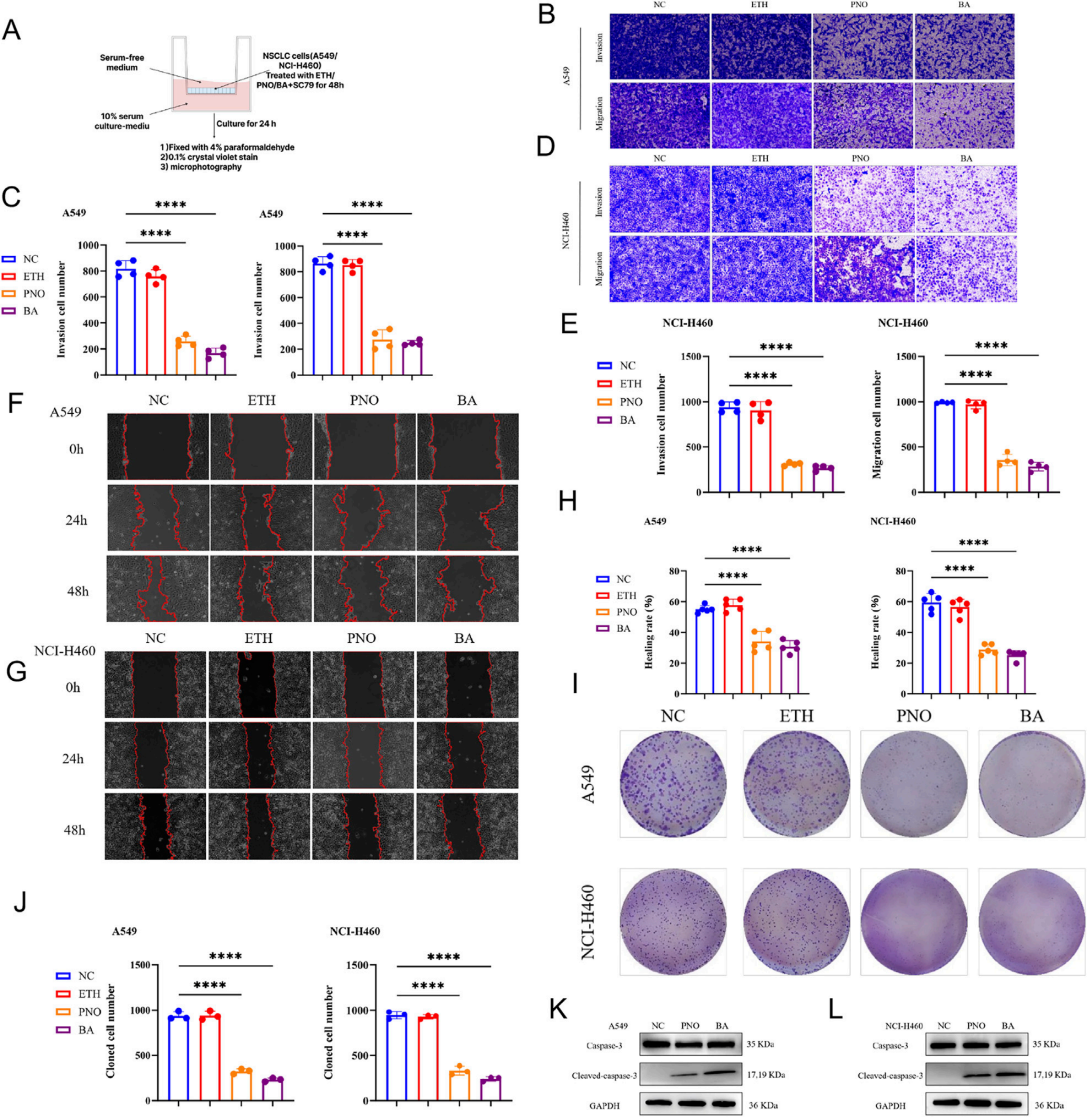
Bornyl acetate inhibits invasion, migration, and clonogenicity in NSCLC cells. **(A)** Transwell assay assessing migration and invasion in A549 and NCI-H460 cells treated with BA (115 μg/mL), PNO (400 μg/mL), or ethanol vehicle. CM served as co-culture control. **(B)** Migratory and invasive A549 cells after 24-h co-culture. Scale bar: 200 µm. **(C)** Quantification of migrating and invading cells. Data represent mean ± SD (n = 3; ****p < 0.0001). **(D)** Migratory and invasive NCI-H460 cells after 24-h co-culture. Scale bar: 200 µm. **(E)** Quantification of migrating and invading cells. Data represent mean ± SD (n = 3; ****p < 0.0001). **(F,G)** Wound healing test in PNO- or BA-treated A549 and NCI-H460 cells at 0, 24, and 48 h (dashed lines indicate wound margins). Scale bar: 200 µm. **(H)** Quantification of wound healing rate. Data represent mean ± SD (n = 3; ****p < 0.0001). **(I)** Representative crystal violet-stained colonies from clonogenic assays. **(J)** Quantification of colony formation. Data represent mean ± SD (n = 3; ****p < 0.0001). **(K,L)** Cleaved/total caspase-3 ratio in PNO- or BA-treated A549 and NCI-H460 cells, respectively.

We further assessed the antitumor effects of BA and PNO on A549 and NCI-H460 cells. Transwell assays showed that BA and PNO significantly inhibited cell invasion and migration versus controls and solvent (ethanol, ETH) (*P* < 0.0001; Figures 3A–E). Wound-healing assays corroborated BA- and PNO-induced suppression of migration (*P* < 0.0001; Figures 3F–H). Colony formation was also markedly reduced by BA and PNO (*P < 0.0001*; Figures 3I,J). Western blotting revealed elevated cleaved-caspase 3 levels in BA- or PNO-treated cells (Figures 3K,L), indicating apoptosis induction. These results demonstrate that BA mediates significant antitumor effects by inhibiting invasion, migration, and clonogenicity.

### 3.4 Bornyl acetate suppresses PI3K/AKT/ABCB1 signaling

**FIGURE 4:**
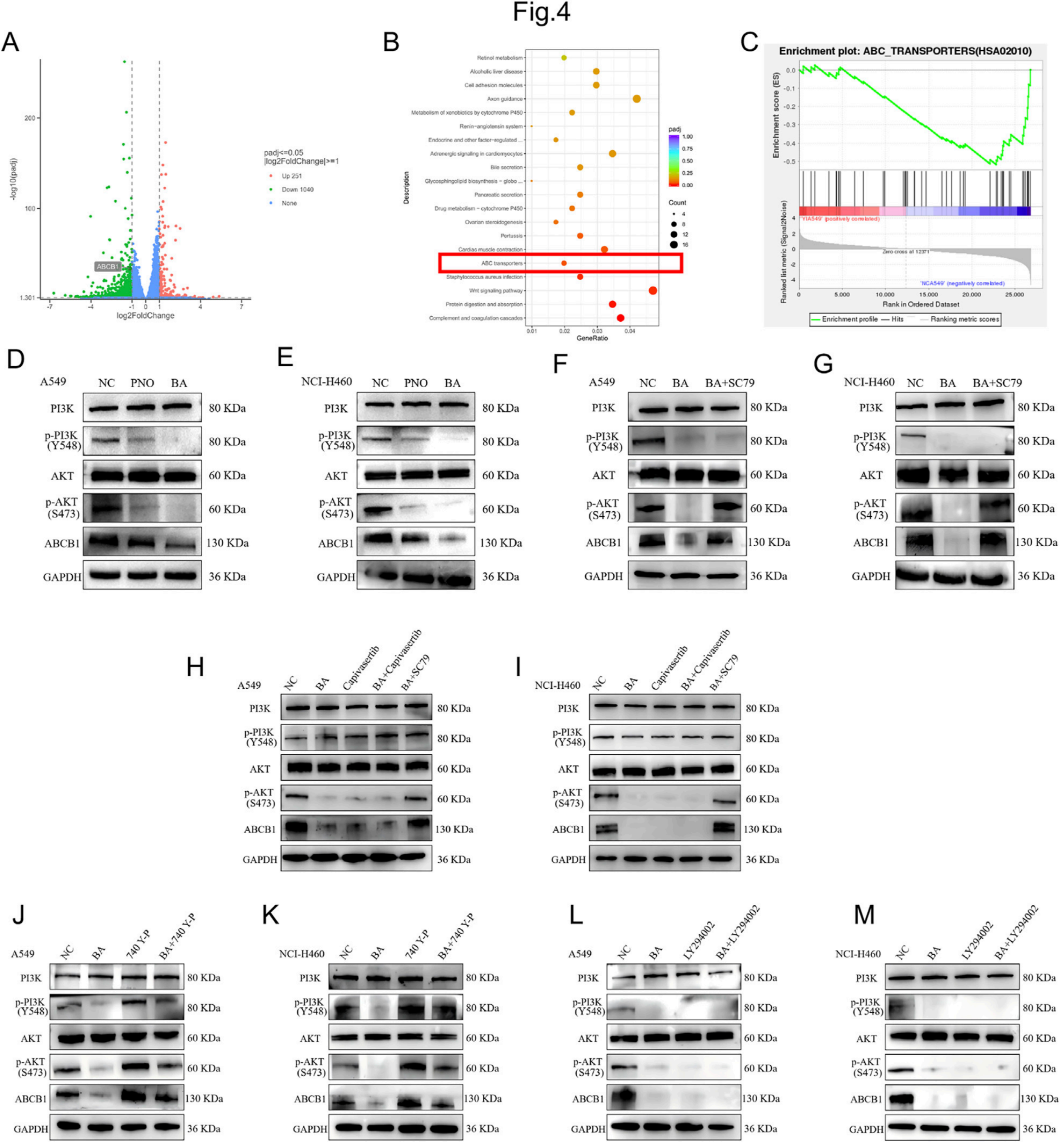
Bornyl acetate suppresses NSCLC proliferation via ABCB1 downregulation. **(A)** Volcano plot of differentially expressed genes in control group vs. BA-treated group A549 cells. **(B)** Top 10 KEGG pathways downregulated following BA treatment. **(C)** GSEA enrichment plot highlighting ABC transporter pathway suppression. **(D,E)** Western blot analysis of PI3K, p-PI3K, AKT, p-AKT, and ABCB1 expression in PNO- or BA-treated A549 and NCI-H460 cells. **(F,G)** Representative Western blots showing expression levels of PI3K, p-PI3K, AKT, p-AKT, and ABCB1 in A549 and NCI-H460 cells treated with BA in combination with the AKT agonist SC79 (10 μM). **(H,I)** Western blot analysis of PI3K, p-PI3K, AKT, p-AKT, and ABCB1 expression in A549 and NCI-H460 cells following treatment with the AKT inhibitor Capivasertib (5 μM) alone, Capivasertib combined with BA, or SC79 combined with BA. **(J,K)** Expression levels of PI3K, p-PI3K, AKT, p-AKT, and ABCB1 as detected by Western blot in A549 and NCI-H460 cells treated with the PI3K agonist 740 Y-P (20 μM) alone or in combination with BA. **(L,M)** Western blot analysis of PI3K, p-PI3K, AKT, p-AKT, and ABCB1 in A549 and NCI-H460 cells treated with the PI3K inhibitor LY294002 (10 μM) alone or in combination with BA.

To elucidate the molecular mechanism of BA in NSCLC, we used RNA sequencing to analyze transcriptome changes in A549 cells before and after BA treatment. RNA sequencing of BA-treated A549 cells identified significant downregulation of ABCB1 (Figures 4A,B). KEGG pathway enrichment implicated ABC transporters in BA’s mechanism (Figure 4C). Previous studies have demonstrated that the PI3K/AKT signaling pathway can activate the expression of the ABCB1 (Wu et al., 2024). Given established PI3K/AKT regulation of ABCB1, Western blot analysis confirmed BA-mediated suppression of p-PI3K(Tyr458), p-AKT (Ser473), and ABCB1 in A549 and NCI-H460 cells (Figures 4D,E).

To pharmacologically reverse the effects of BA, we applied the specific AKT agonist SC79 (10 μM). Western blot analysis was performed to evaluate the expression levels of PI3K, p-PI3K, AKT, p-AKT, and ABCB1 in A549 and NCI-H460 cells treated with SC79 in combination with BA. The results indicated that co-treatment with SC79 and BA led to an upward trend in p-AKT and ABCB1 expression (Figures 4F,G).

To further examine the involvement of the PI3K/AKT pathway in BA-induced ABCB1 suppression, we treated A549 and NCI-H460 cells with the AKT inhibitor Capivasertib (5 μM), either alone or in combination with BA, along with the AKT agonist SC79 plus BA. Western blot analysis revealed that Capivasertib alone downregulated ABCB1 expression, while its combination with BA produced a synergistic inhibitory effect. Conversely, activation of AKT by SC79 effectively reversed the suppressive effect of BA (Figures 4H,I).

In parallel, we used the PI3K agonist 740 Y-P (20 μM), both alone and in combination with BA. Western blot results demonstrated that 740 Y-P alone significantly elevated the protein levels of p-PI3K, p-AKT, and ABCB1. Moreover, PI3K activation markedly counteracted the BA-mediated downregulation of p-AKT and ABCB1 under combined treatment (Figures 4J,K).

Additionally, treatment with the PI3K inhibitor LY294002 (10 μM), either alone or together with BA, resulted in significant reduction of p-PI3K, p-AKT, and ABCB1 levels. The combination of LY294002 and BA synergistically enhanced this inhibitory effect, indicating that PI3K inhibition mimics the cellular actions of BA (Figures 4L,M). In conclusion, BA modulates the PI3K/AKT signaling axis leading to downregulation of ABCB1, thereby exerting its functional effects.

Rescue experiments with AKT agonist SC79 partially reversed BA-induced inhibition of invasion (*P < 0.001*), migration (*P < 0.001*), and clonogenicity (*P < 0.001*) (Supplementary Figure S1A–H). Western blot was used to detect the expression levels of PI3K, p-PI3K, AKT, p-AKT and ABCB1 in A549 and NCI-H460 cells treated with different doses of AKT agonist (SC79). The results showed increased expression of p-AKT and ABCB1, indicating successful activation of AKT by SC79 (Supplementary Figure S1I,J). Caspase3 and cleaved-caspase 3 expression was also examined, and cleaved-caspase 3 expression was elevated in the presence of SC79 and BA (Supplementary Figure S1K,L). These demonstrates functional dependence on PI3K/AKT signaling for BA’s anti-tumor effects.

### 3.5 Bornyl acetate inhibits NSCLC tumor growth *in vivo*

**FIGURE 5:**
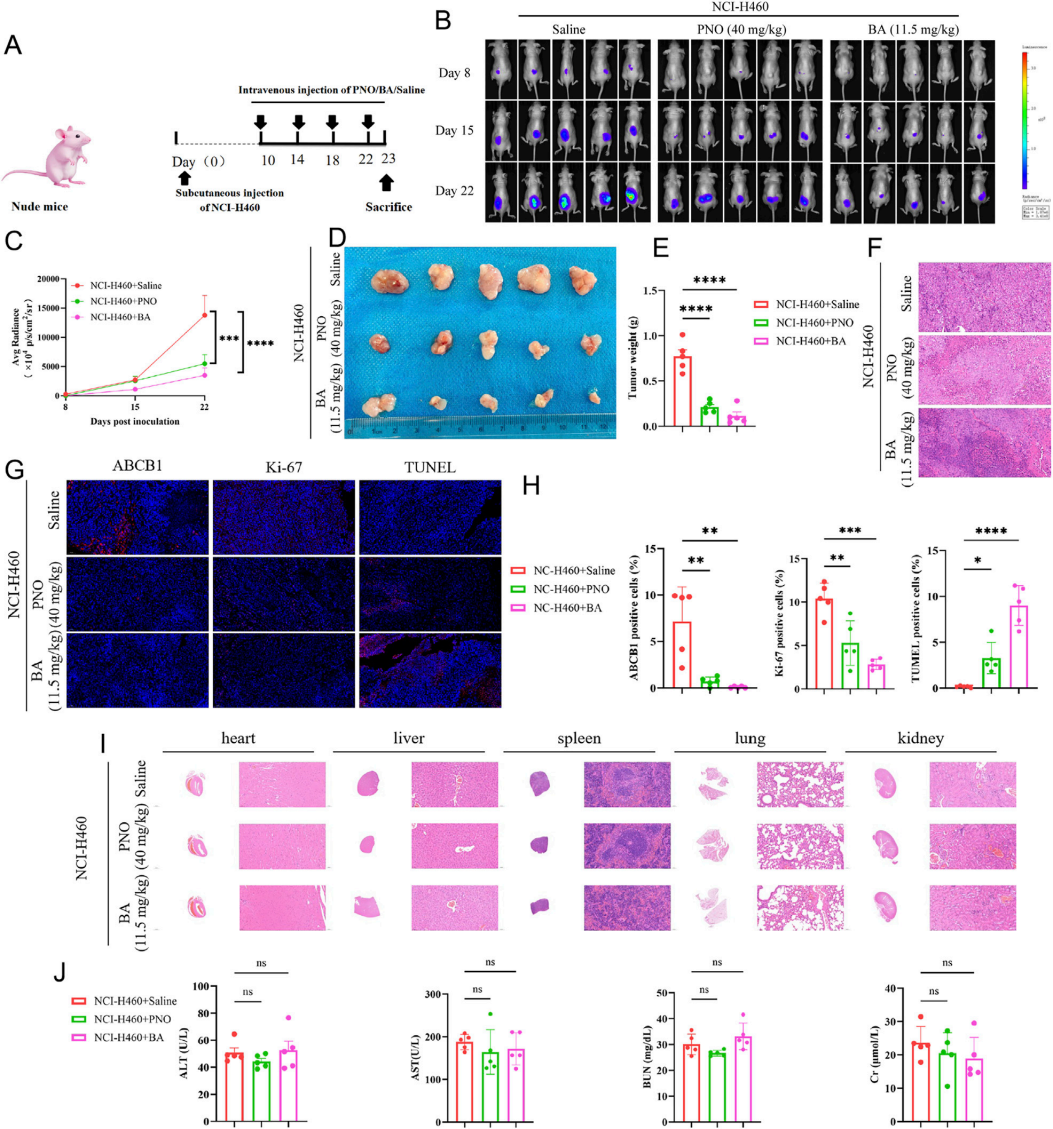
Borneol acetate inhibits NCI-H460 tumor growth *in vivo.* **(A)** Experimental schema: Nude mice received subcutaneous injections of 2 × 10^6^ NCI-H460-luciferase cells. Vehicle, PNO, or BA were administered via tail vein. Tumor progression was monitored by bioluminescence imaging (BLI); tumors and organs were harvested terminally. **(B)** BLI pseudocolor images on days 8, 15, and 22. **(C)** Quantified fluorescence intensity. Data represent mean ± SD (*p < 0.001, *p* < 0.0001). **(D)** Excised tumors. Reduced BA group volume demonstrates tumor suppression. **(E)** Tumor weight quantification (p < 0.0001). **(F)** H&E-stained tumor sections. **(G)** Immunofluorescence staining of tumor sections: ABCB1 (blue, resistance marker), Ki67 (green, proliferation marker), TUNEL (red, apoptosis marker). Scale bar: 50 µm. **(H)** Quantified immunofluorescence intensity. Data represent mean ± SD (**p < 0.01, ***p < 0.001, ****p < 0.0001). **(I)** H&E staining of liver, spleen, lung, and kidney sections. **(J)** Liver/kidney function indices (*ns p* > 0.05).

To evaluate the *in vivo* antitumor efficacy of BA and PNO, 2 × 10^6^ NCI-H460-luciferase cells were subcutaneously injected into the flanks of nude mice. Subsequently, mice received intravenous injections of either 11.5 mg/kg BA, 40 mg/kg PNO, or saline (control group; Figure 5A). Tumor progression was monitored closely using BLI, and tumor fluorescence intensity was recorded. The results demonstrated a significant reduction in fluorescence intensity in both BA- and PNO-treated groups compared to the saline control group (*P* < 0.0001; Figures 5B,C). At the experimental endpoint, tumors were excised for histological and immunofluorescence analyses. BA and PNO treatment significantly reduced both tumor size and tumor weight (Figures 5D,E). Hematoxylin and eosin (H&E) staining revealed a greater number of necrotic foci within tumor tissues in the treatment groups compared to the control group (Figure 5F). Immunofluorescence staining for ABCB1, Ki-67, and TUNEL showed that BA treatment downregulated ABCB1 expression, decreased Ki-67 expression, and increased TUNEL expression (*P* < 0.0001; Figures 5G,H). H&E staining of major organs (heart, liver, spleen, lung, and kidney) revealed relatively normal tissue architecture, indicating no obvious histopathological toxicity (Figure 5I). Assessment of liver and kidney function showed no increase in alanine aminotransferase (ALT) or aspartate aminotransferase (AST) levels in BA- or PNO-treated groups. While blood urea nitrogen (BUN) levels were slightly elevated, this change was not statistically significant. Creatinine (Cr) levels remained unchanged (Figure 5J).

**FIGURE 6:**
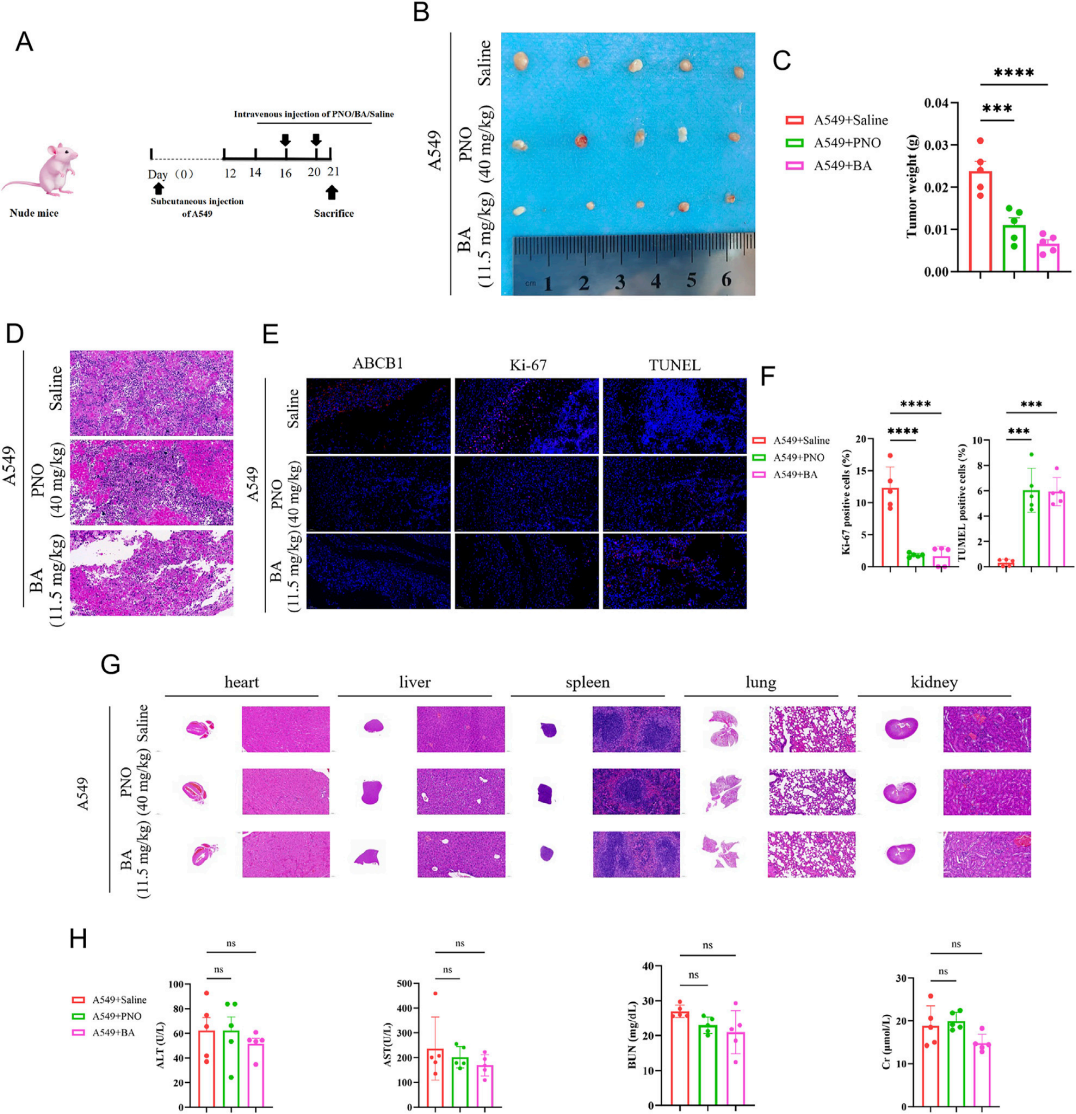
Borneol acetate inhibits A549 tumor growth *in vivo*. **(A)** Experimental schema: Nude mice received subcutaneous injections of 2 × 10^6^ A549-luciferase cells. Treatments identical to Figure 5A. **(B)** Excised tumors. Reduced BA group volume indicates tumor suppression. **(C)** Tumor weight quantification (****p < 0.0001). **(D)** H&E-stained tumor sections. **(E,F)** Immunofluorescence staining of tumor sections (markers as in Figure 5G). Scale bar: 50 µm. **(G)** H&E staining of major organs. **(H)** Liver/kidney function indices (ALT, AST, BUN, Cr; *ns: p* > 0.05).

**FIGURE 7:**
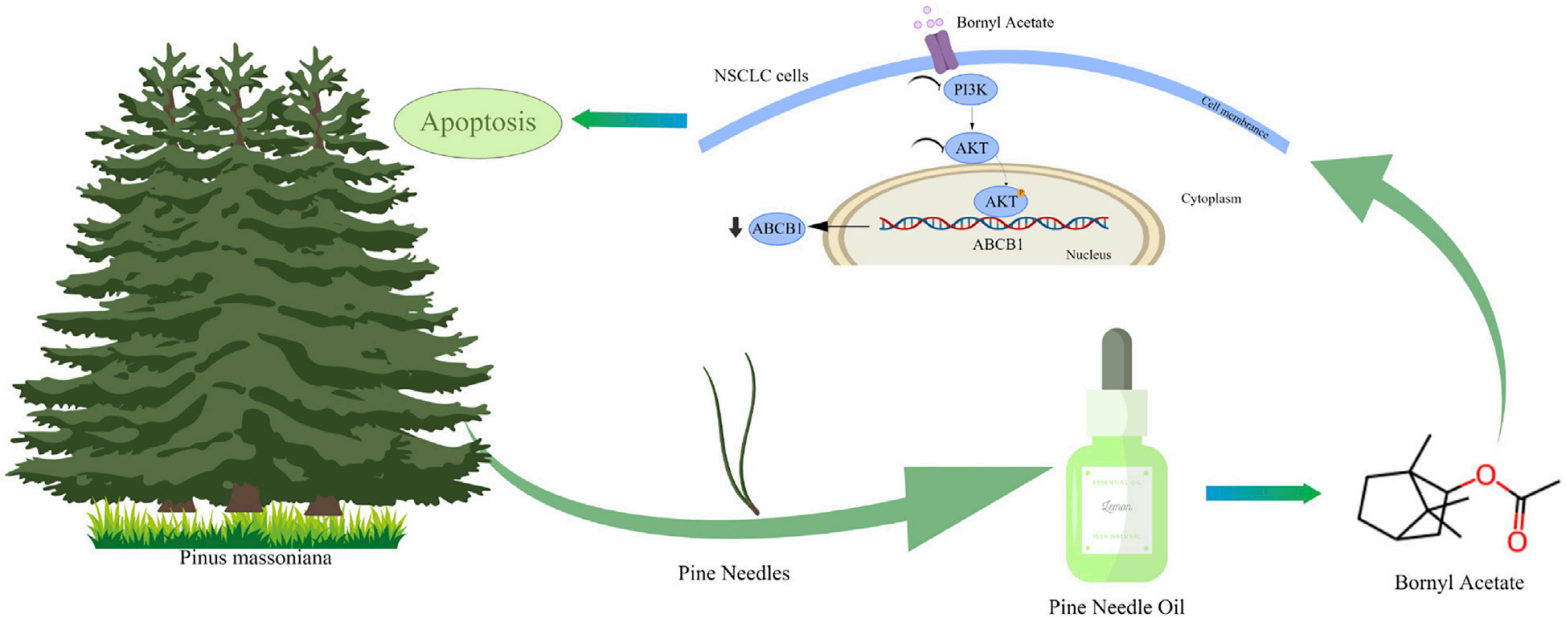
Graphical abstract. The active constituent of pine needle oil, borneol acetate, exerts potent anti-NSCLC effects by targeting the PI3K/AKT/ABCB1 axis.

Consistent results were observed in the A549 xenograft models. BA and PNO treatment significantly reduced tumor size and weight (*P* < 0.0001; Figures 6A–C). H&E staining again indicated increased necrotic foci in tumors from treated mice relative to controls (Figure 6D). Immunofluorescence analysis confirmed that BA treatment downregulated ABCB1 expression, decreased Ki-67 expression, and increased TUNEL expression (*P* < 0.0001; Figures 6E,F). H&E examination of vital organs (heart, liver, spleen, lungs, and kidneys) showed relatively normal structures and no significant histopathological toxicity (Figure 6G). Liver and kidney function tests revealed no increase in ALT or AST levels, a slight but statistically non-significant decrease in BUN levels (Note: Direction change to match Figure 6H description), and no increase in Cr levels in the BA- or PNO-treated groups (Figure 6H). These findings collectively demonstrate that BA and PNO effectively inhibit NSCLC growth and has no significance toxicity *in vivo*. Our findings demonstrated that BA exerts potent anti-NSCLC effects by targeting the PI3K/AKT/ABCB1 axis (Figure 7).

## 4 Discussion

NSCLC remains a formidable clinical challenge due to the frequent development of multidrug resistance (MDR), often driven by the overexpression of efflux transporters like ABCB1/P-glycoprotein. Natural products offer a rich source of multi-target agents to overcome MDR. Here, we identify BA, a key monoterpenoid component of PNO, as a potent inhibitor of NSCLC progression. We demonstrate that BA suppresses proliferation, invasion, migration, and induces apoptosis *in vitro*, and inhibits tumor growth *in vivo*. Critically, through comprehensive pharmacological modulation (using PI3K inhibitor LY294002, activator 740Y-P, AKT activator SC79 and inhibitor Capivasertib), we establish that BA exerts these effects primarily through inhibition of the PI3K/AKT/ABCB1 signaling axis. Although targeted therapies (e.g., EGFR-TKIs, ALK inhibitors) (Cooper et al., 2022) and immunotherapies (e.g., PD-1/PD-L1 inhibitors) (Chen et al., 2020) have made progress in recent years, the development of drug resistance has severely limited their clinical efficacy. Resistance mechanisms driven by genetic alterations (such as MET amplification), epigenetic modifications, and tumor microenvironment adaptation greatly reduce therapeutic efficacy (Lu et al., 2021). Therefore, there is an urgent need for new drugs that can overcome multidrug resistance. Natural products are an important source of such drugs, exemplified by paclitaxel (Pi et al., 2022) and vinorelbine (Ohe et al., 2007) in current NSCLC treatment regimens.

Previous studies have shown that bornyl acetate (BA), a key monoterpenoid in PNO, exhibits antitumor activity in liver cancer and breast cancer models by inhibiting proliferation and angiogenesis through inhibition of PI3K/AKT and MAPK/ERK pathways (Chen et al., 2015; Ren et al., 2018). However, its role in NSCLC has not been explored. This study fills this critical gap and demonstrates that BA, as a key active component of PNO, exerts a powerful antitumor effect in NSCLC. BA significantly inhibited viability, invasion, migration and colony formation of A549 and NCI-H460 cells, and induced apoptosis through caspase-3 cleavage.

The PI3K/AKT pathway is a central driver of tumor survival, proliferation, and treatment resistance in NSCLC (Shi et al., 2022; McDaid et al., 2024). Crucially, this pathway regulates ATP-binding cassette (ABC) transporters, including ABCB1 (P-glycoprotein) (Wu et al., 2024), which mediate chemotherapy resistance through drug efflux (Skinner et al., 2023). Consistent with previous reports, we demonstrate that activation of PI3K/AKT promotes ABCB1 expression. Our RNA-seq analysis showed BA downregulates ABCB1 gene expression, while Western blotting showed simultaneous suppression of p-PI3K, p-AKT, and ABCB1 protein levels. Functional rescue experiments using the AKT agonist SC79 provided initial confirmation of this axis’s involvement. To further establish causality, we utilized a panel of pharmacological tools. Notably, the PI3K inhibitor LY294002 mimicked the effect of BA by reducing p-AKT and ABCB1 levels, whereas the PI3K activator 740Y-P markedly attenuated BA-induced downregulation of these proteins. These findings offer direct functional evidence that PI3K acts as a key upstream mediator of BA’s action. Moreover, the AKT inhibitor Capivasertib alone downregulated ABCB1, and its combination with BA produced a synergistic effect, underscoring the central role of AKT in this regulatory cascade.

An interesting comparison emerged from the efficacy profiles of PNO and BA. The IC_50_ value for PNO was approximately 500 μg/mL, compared to ∼100 μg/mL for BA. Given that BA constitutes only 3.1% of PNO, its theoretical IC_50_—should it be the sole active component—would be around 15.5 μg/mL (500 μg/mL × 3.1% = 15.5 μg/mL). The experimentally determined IC_50_ of BA is substantially higher than this calculated value, suggesting that although BA is the most potent constituent identified, it is likely not the only active compound in PNO. The significant tumor suppression observed with PNO *in vivo* may result from additive or synergistic interactions between BA and other components, such as β-pinene, camphene, or other monoterpenes, which have documented anticancer properties (Kamran et al., 2022). This discrepancy underscores the complexity of natural extracts and implies that whole extracts may possess therapeutic benefits over single compounds. Further studies are needed to systematically examine the interactions between BA and other major constituents of PNO to fully elucidate its composition–activity relationship.

Despite these compelling results, our study has several limitations. First, although pharmacological evidence strongly supports the involvement of the PI3K/AKT/ABCB1 pathway, genetic gain- and loss-of-function studies are required to establish definitive causality. Second, while we observed ABCB1 downregulation at the protein level, we did not functionally assess reduced pump activity using methods such as calcein-AM accumulation assays. Third, the *in vitro* models used (A549 and NCI-H460) do not directly represent EGFR-mutant or ALK-fusion-positive contexts, where overcoming TKI resistance is most clinically relevant. Fourth, the semi-quantitative nature of our GC-MS analysis and the unexamined bioavailability of BA highlight important areas for future pharmaceutical development. Finally, although BA induced apoptosis, its potential effects on other cell death mechanisms (e.g., autophagy) or DNA damage response remain unexplored.

Although pharmacological modulators of PI3K (LY294002 and 740Y-P) robustly demonstrate that BA suppresses the PI3K/AKT/ABCB1 pathway, this approach does not constitute direct target validation. Specifically, our study lacks direct measurement of PI3K kinase activity to conclusively prove that BA inhibits PI3K enzymatically. Thus, while our data strongly implicate PI3K as an upstream effector, we cannot exclude the possibility that BA inhibits AKT phosphorylation via an alternative target. Future studies using direct enzymatic assays and genetic manipulations (e.g., PI3K overexpression or siRNA knockdown) will be essential to confirm causality and account for potential off-target effects.

Beyond mechanistic insights, the translational potential of a new compound must be rigorously assessed. Although tools such as the AKT agonist SC79 and the PI3K activator.

740 Y-P were valuable for pathway validation, they are unsuitable for therapeutic use due to known off-target effects (e.g., SC79’s induction of nitric oxide synthesis). Moreover, the clinical development of BA faces considerable challenges. Critical parameters including its bioavailability, pharmacokinetic profile, optimal formulation, and safety window in humans remain largely unknown. These aspects represent essential prerequisites that must be thoroughly investigated before BA can be seriously considered for therapeutic applications.

## 5 Conclusion

In conclusion, our study offers robust pharmacological evidence that BA, a principal bioactive constituent of PNO, inhibits NSCLC progression by targeting the PI3K/AKT signaling pathway and downregulating ABCB1 expression. The consistent anti-tumor effects observed across both *in vitro* and *in vivo* models strongly supports this mechanism. These results not deepen the understanding of BA’s anti-cancer properties, but also establish a basis for further research into ABC transporter regulation in therapy-resistant NSCLC. Future studies employing genetic manipulations and functional transporter assays will be crucial to fully delineate this pathway and evaluate its therapeutic relevance. This work provides the first evidence of the effectiveness of BA in NSCLC and elucidates a novel mechanism for reversing resistance. Our findings position BA as a promising combination therapy candidate with the potential to enhance efficacy while reducing toxicity in drug-resistant NSCLC. Future studies will need to optimize delivery systems and assess the efficacy of BA in combination with standard chemotherapy drugs.

## Data Availability

The original contributions presented in the study are included in the article/Supplementary Material, further inquiries can be directed to the corresponding authors.
