# Correction: The active constituent of pine needle oil, bornyl acetate, suppresses NSCLC progression by inhibiting the PI3K/AKT/ABCB1 signaling axis

**DOI:** 10.3389/fphar.2025.1730938

**Published:** 2025-11-13

**Authors:** Jieheng Wu, Jiaming Ren, Lu Wang, Zeyang Yang, Xuanyin Wang, Ziyi Tu, Xinlei Liu, Ye Wang, Yaxuan Cao, Xu Zhu, Long Li, Maoqin Lu, Ying Zhang, Jinyi Wu, Yu Cao

**Affiliations:** 1 Department of Immunology, Guizhou Medical University, Guiyang, China; 2 Tumor Immunotherapy Technology Engineering Research Center of Guizhou Medical University, Guizhou Medical University, Guiyang, China; 3 School of Public Health, the key Laboratory of Environmental Pollution Monitoring and Disease Control, Ministry of Education, Guizhou Medical University, Guiyang, China; 4 Department of Anatomy, Guizhou Medical University, Guiyang, China; 5 Guizhou Prenatal Diagnsis Center, The Affiliated Hospital of Guizhou Medical University, Guiyang, China; 6 Department of Nursing, Guiyang Healthcare Vocational University, Guiyang, China; 7 School of Clinical Medicine, Guizhou Medical University, Guiyang, China; 8 Department of Thoracic Surgery, The Affiliated Hospital of Guizhou Medical University, Guiyang, China

**Keywords:** bornyl acetate, NSCLC, PI3K/AKT, ABCB1, tumor progression

There was a mistake in Figure 5B as published. During a recent review of the figures, we discovered a duplication in Figure 5B, specifically in the panel showing the *in vivo* imaging results of the saline control group on Day 22. The images for the third and fourth mice were inadvertently repeated during the figure preparation process. We have carefully rechecked the original data and confirmed that the error occurred during figure assembly and layout, and not in the experimental data itself. The underlying findings and conclusions of the study remain valid and unaffected. The corrected Figure 5 appears below.

**FIGURE 5:**
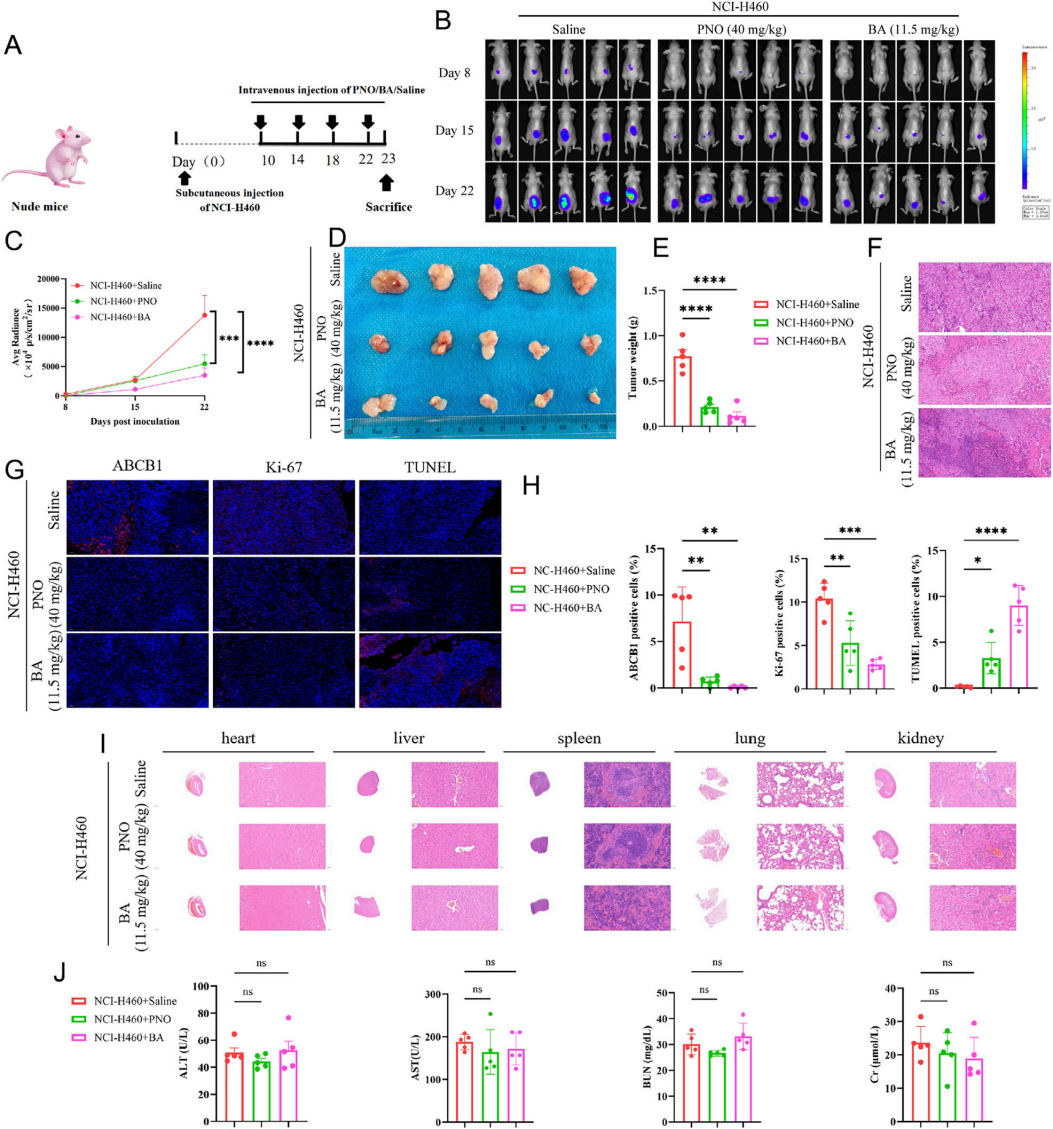
Borneol acetate inhibits NCI-H460 tumor growth in vivo. **(A)** Experimental schema: Nude mice received subcutaneous injections of 2 × 10^6^ NCI-H460-luciferase cells. Vehicle, PNO, or BA were administered via tail vein. Tumor progression was monitored by bioluminescence imaging (BLI); tumors and organs were harvested terminally. **(B)** BLI pseudocolor images on days 8, 15, and 22. **(C)** Quantified fluorescence intensity. Data represent mean ± SD (*p < 0.001, p < 0.0001). **(D)** Excised tumors. Reduced BA group volume demonstrates tumor suppression. **(E)** Tumor weight quantification (p < 0.0001). **(F)** H&E-stained tumor sections. **(G)** Immunofluorescence staining of tumor sections: ABCB1 (blue, resistance marker), Ki67 (green, proliferation marker), TUNEL (red, apoptosis marker). Scale bar: 50 µm. **(H)** Quantified immunofluorescence intensity. Data represent mean ± SD (**p < 0.01, ***p < 0.001, ****p < 0.0001). **(I)** H&E staining of liver, spleen, lung, and kidney sections. **(J)** Liver/kidney function indices (ns p > 0.05).

The original article has been updated.

