# Supplementary figures and images for: The active constituent of pine needle oil, bornyl acetate, suppresses NSCLC progression by inhibiting the PI3K/AKT/ABCB1 signaling axis
Source: Front Pharmacol. 2025 Sep 23;16:1653461. doi: 10.3389/fphar.2025.1653461 (PMC12500681; doi:10.3389/fphar.2025.1653461)

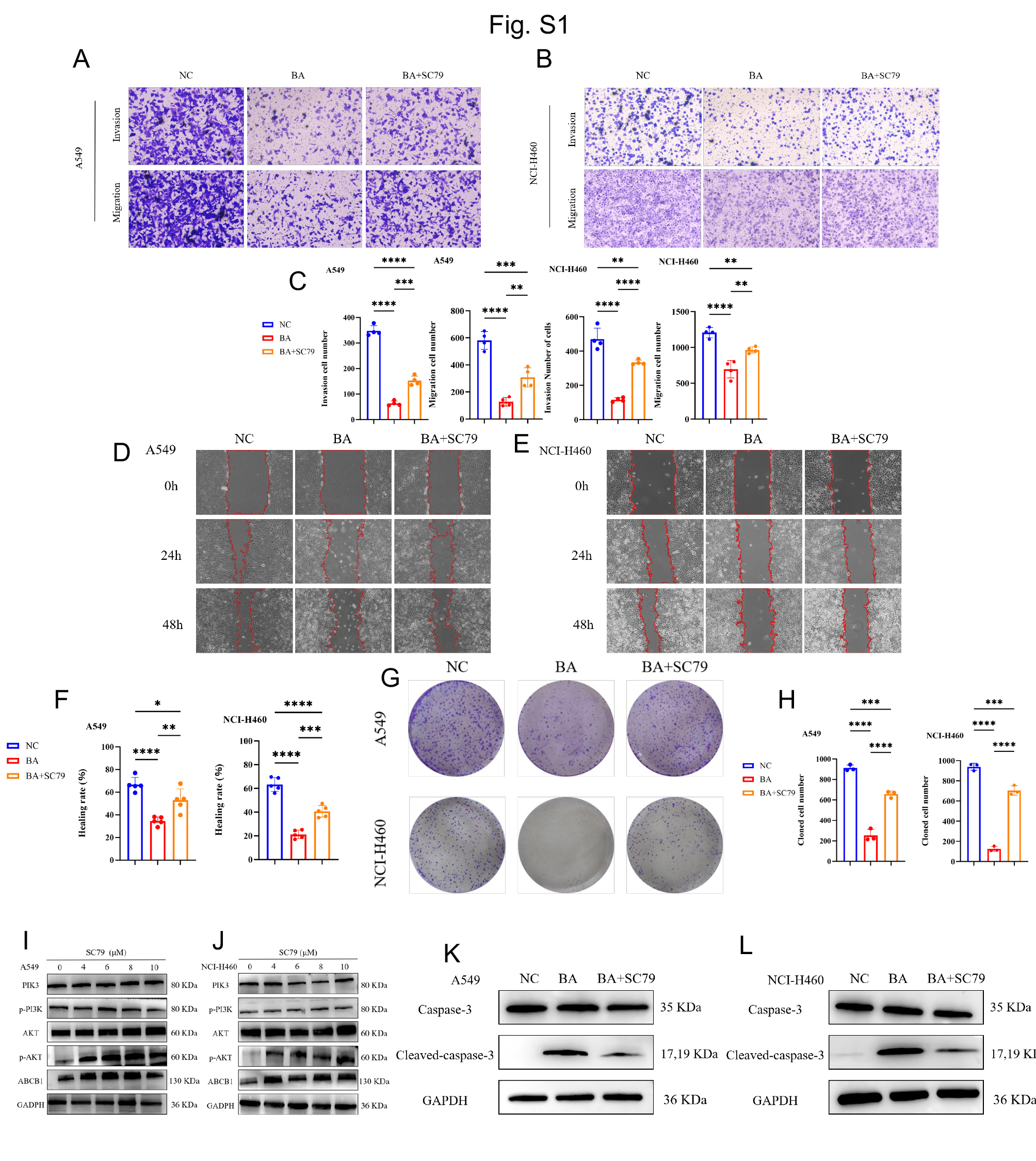

Supplement: Supplementary file 1 [file Image1.tif]
