# Supplementary material for: The active constituent of pine needle oil, bornyl acetate, suppresses NSCLC progression by inhibiting the PI3K/AKT/ABCB1 signaling axis
Source: Front Pharmacol. 2025 Sep 23;16:1653461. doi: 10.3389/fphar.2025.1653461 (PMC12500681; doi:10.3389/fphar.2025.1653461)
Supplement: Supplementary file 2 [file DataSheet1.pdf]

Fig3.K

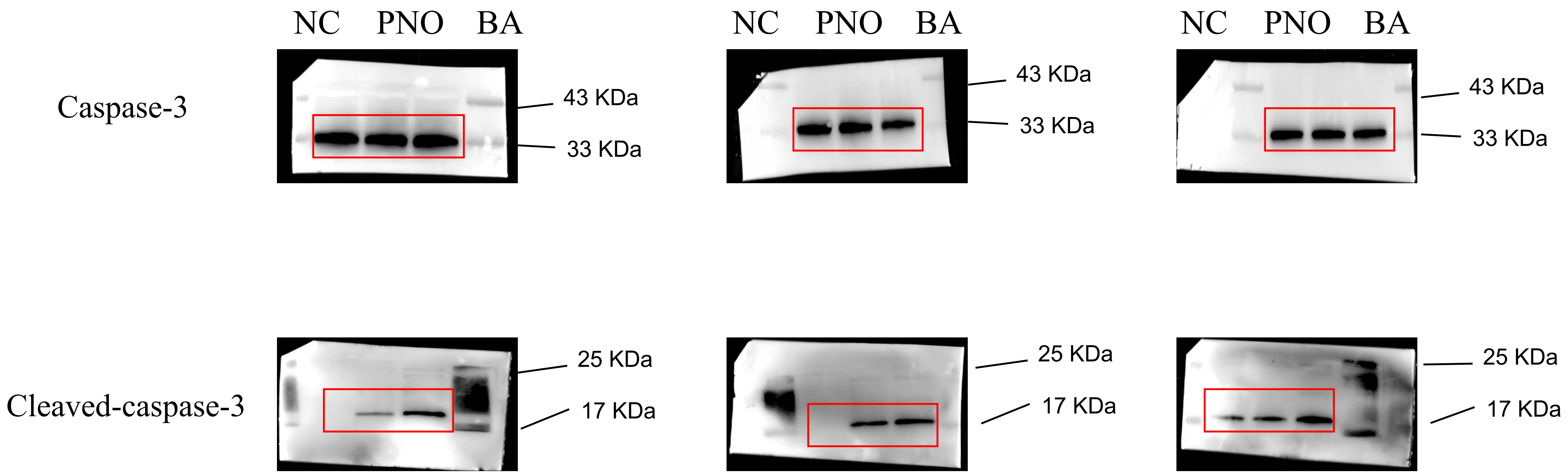
NC
PNO
BA
Caspase-3
43 KDa
33 KDa
NC
PNO
BA
43 KDa
33 KDa
NC
PNO
BA
43 KDa
33 KDa
Cleaved-caspase-3
25 KDa
17 KDa
25 KDa
17 KDa
25 KDa
17 KDa

Fig3.L

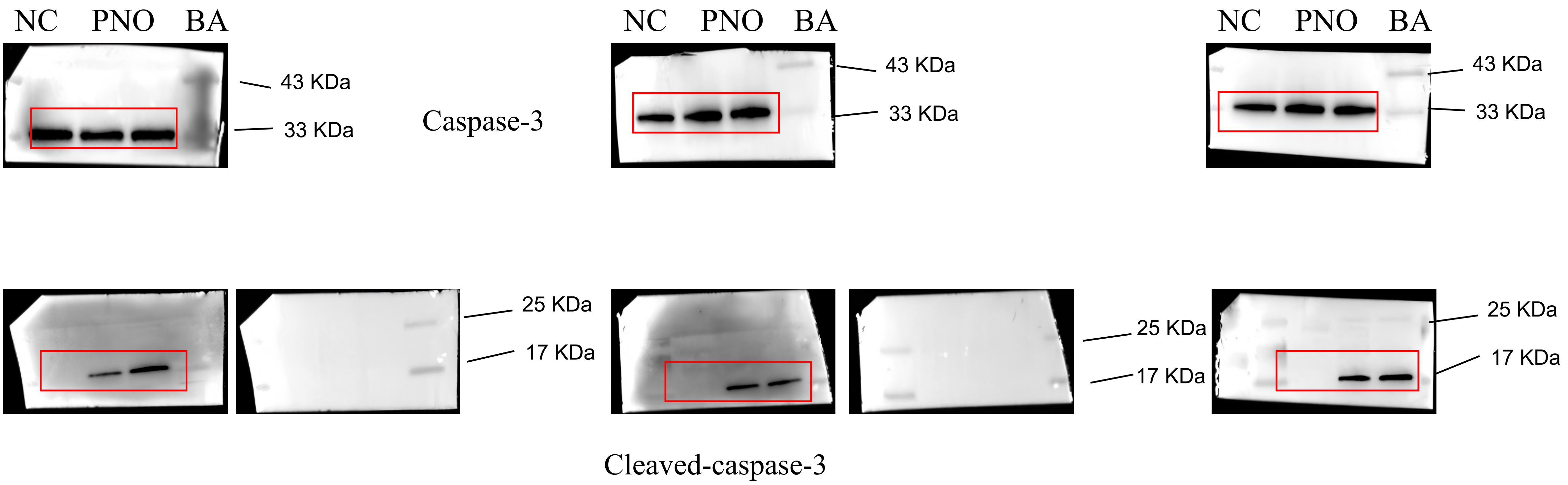
NC
PNO
BA
43 KDa
33 KDa
Caspase-3
NC
PNO
BA
43 KDa
33 KDa
NC
PNO
BA
43 KDa
33 KDa
25 KDa
17 KDa
25 KDa
17 KDa
25 KDa
17 KDa
Cleaved-caspase-3

Fig4.D

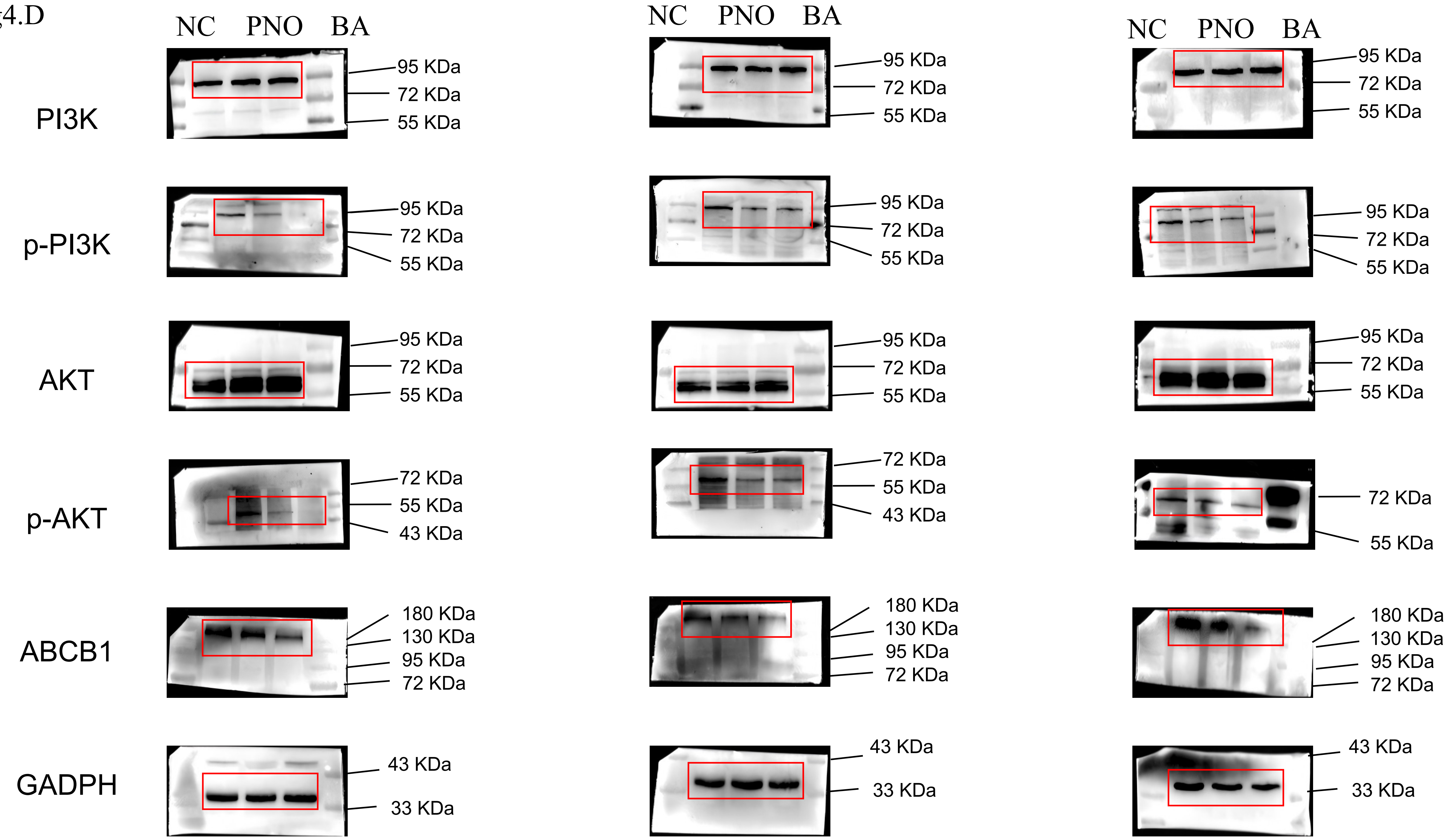

NC PNO BA
NC PNO BA
NC PNO BA
PI3K
p-PI3K
AKT
p-AKT
ABCB1
GADPH
95 KDa
72 KDa
55 KDa
43 KDa
33 KDa
180 KDa
130 KDa

Fig3.E

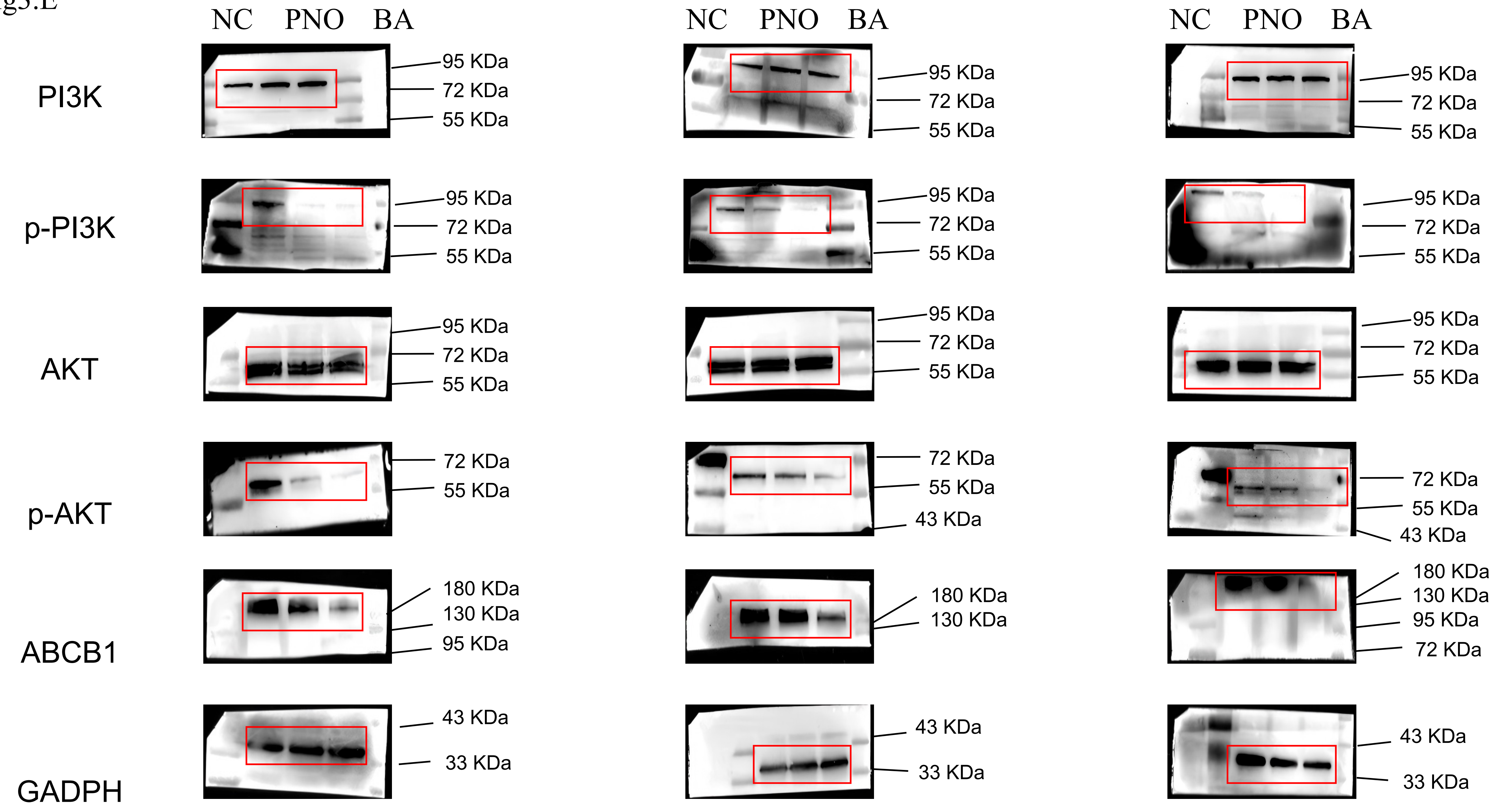

NC PNO BA
NC PNO BA
NC PNO BA
PI3K
p-PI3K
AKT
p-AKT
ABCB1
GADPH
95 KDa
72 KDa
55 KDa
43 KDa
33 KDa
180 KDa
130 KDa

Fig4. F

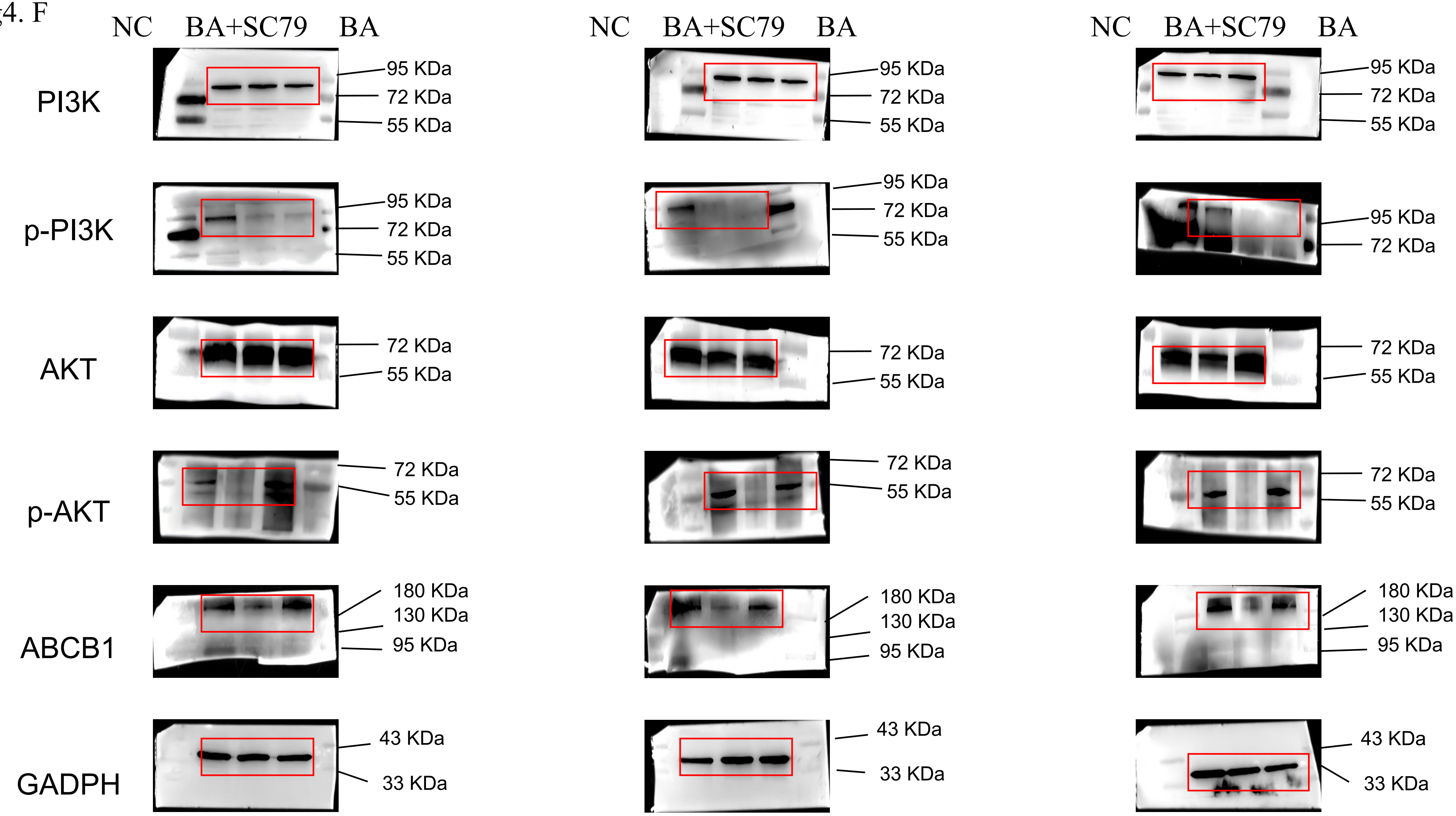
NC BA+SC79 BA
NC BA+SC79 BA
NC BA+SC79 BA
PI3K
p-PI3K
AKT
p-AKT
ABCB1
GADPH
180 KDa
130 KDa
95 KDa
72 KDa
55 KDa
43 KDa
33 KDa

Fig4. G

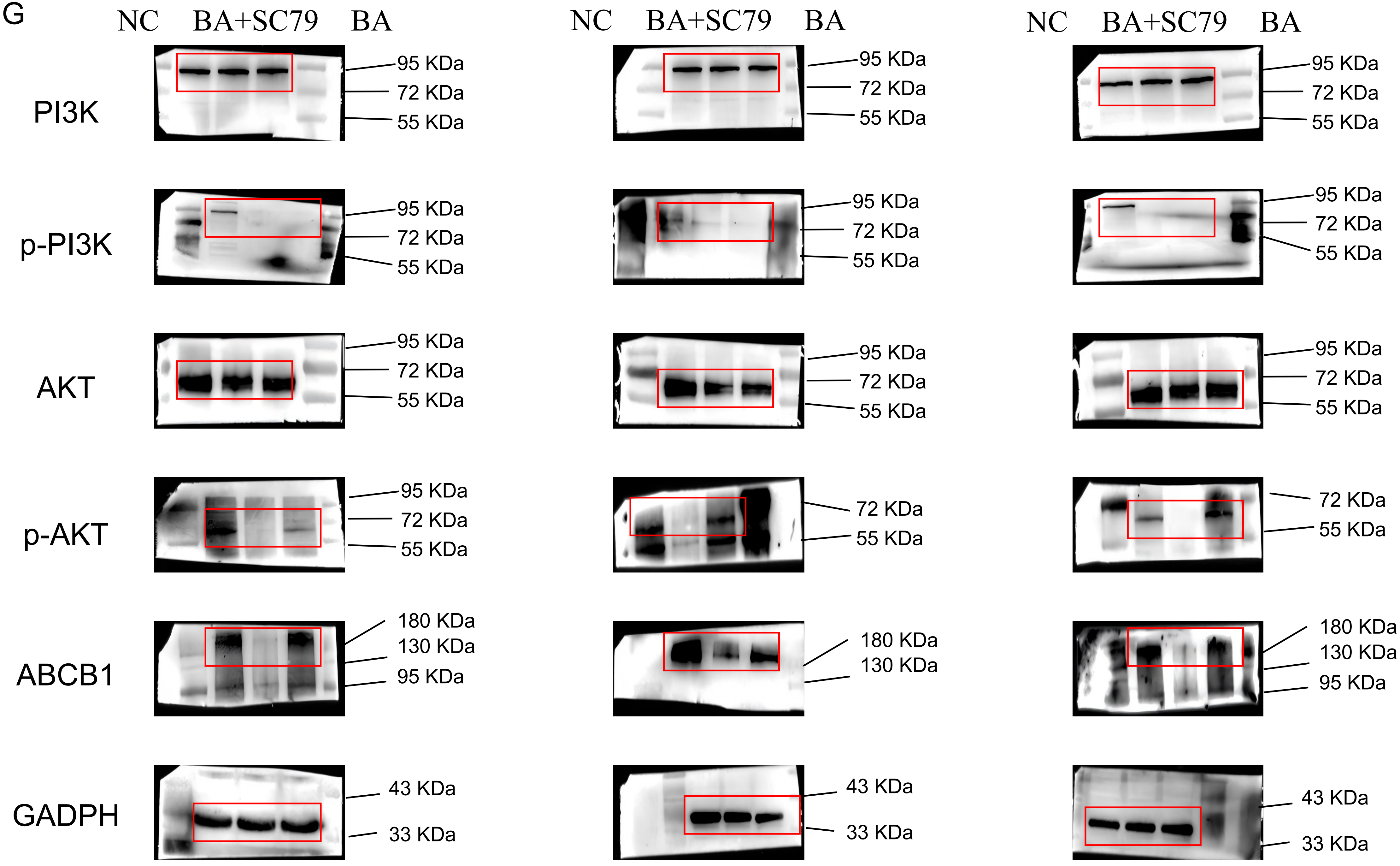
NC
BA+SC79
BA
NC
BA+SC79
BA
NC
BA+SC79
BA
PI3K
95 KDa
72 KDa
55 KDa
p-PI3K
95 KDa
72 KDa
55 KDa
AKT
95 KDa
72 KDa
55 KDa
p-AKT
95 KDa
72 KDa
55 KDa
ABCB1
180 KDa
130 KDa
95 KDa
GADPH
43 KDa
33 KDa

Fig4. H

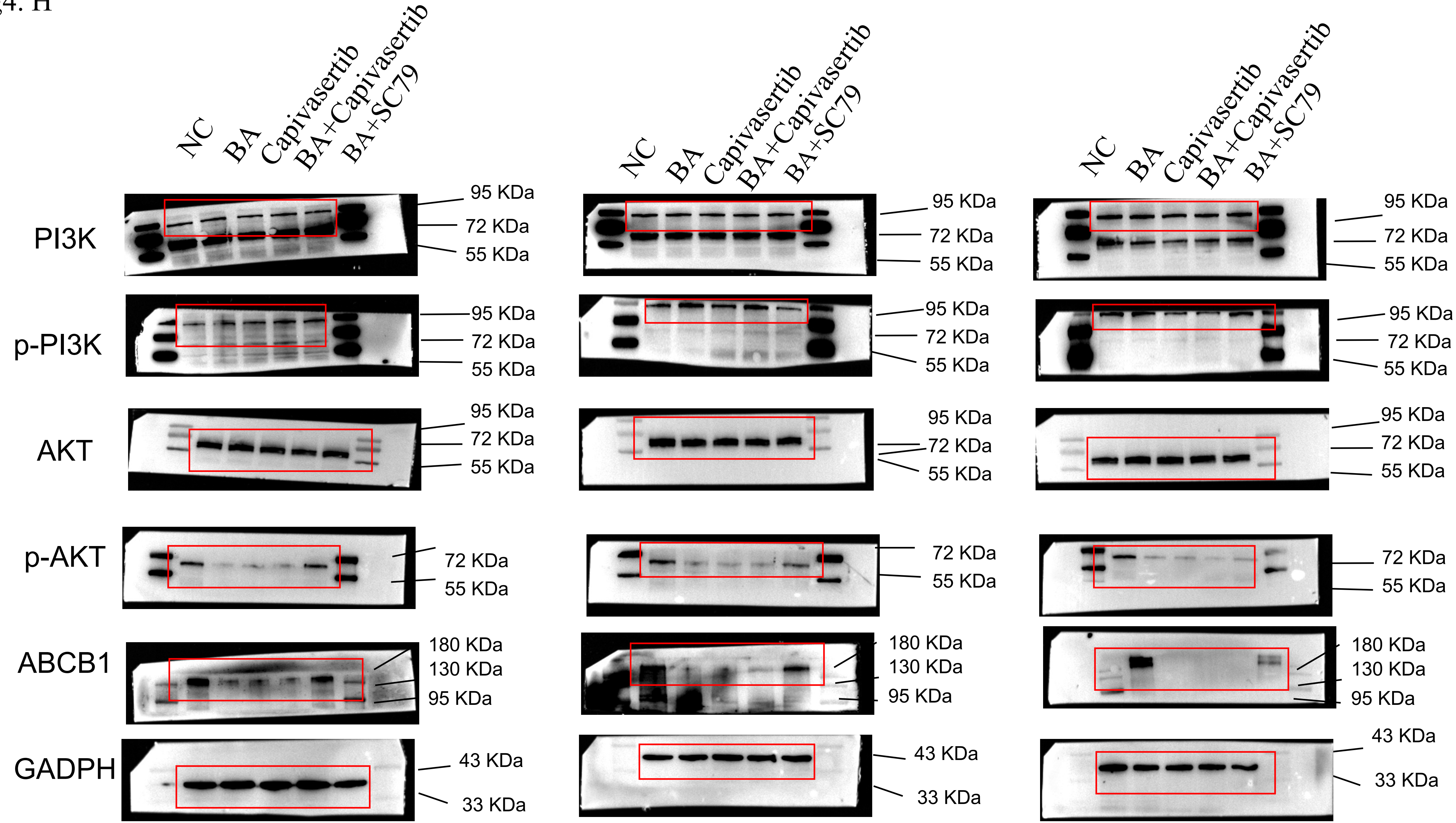
NC
BA
Capivasertib
BA+Capivasertib
BA+SC79
PI3K
p-PI3K
AKT
p-AKT
ABCB1
GADPH
180 KDa
130 KDa
95 KDa
72 KDa
55 KDa
43 KDa
33 KDa

Fig4. I

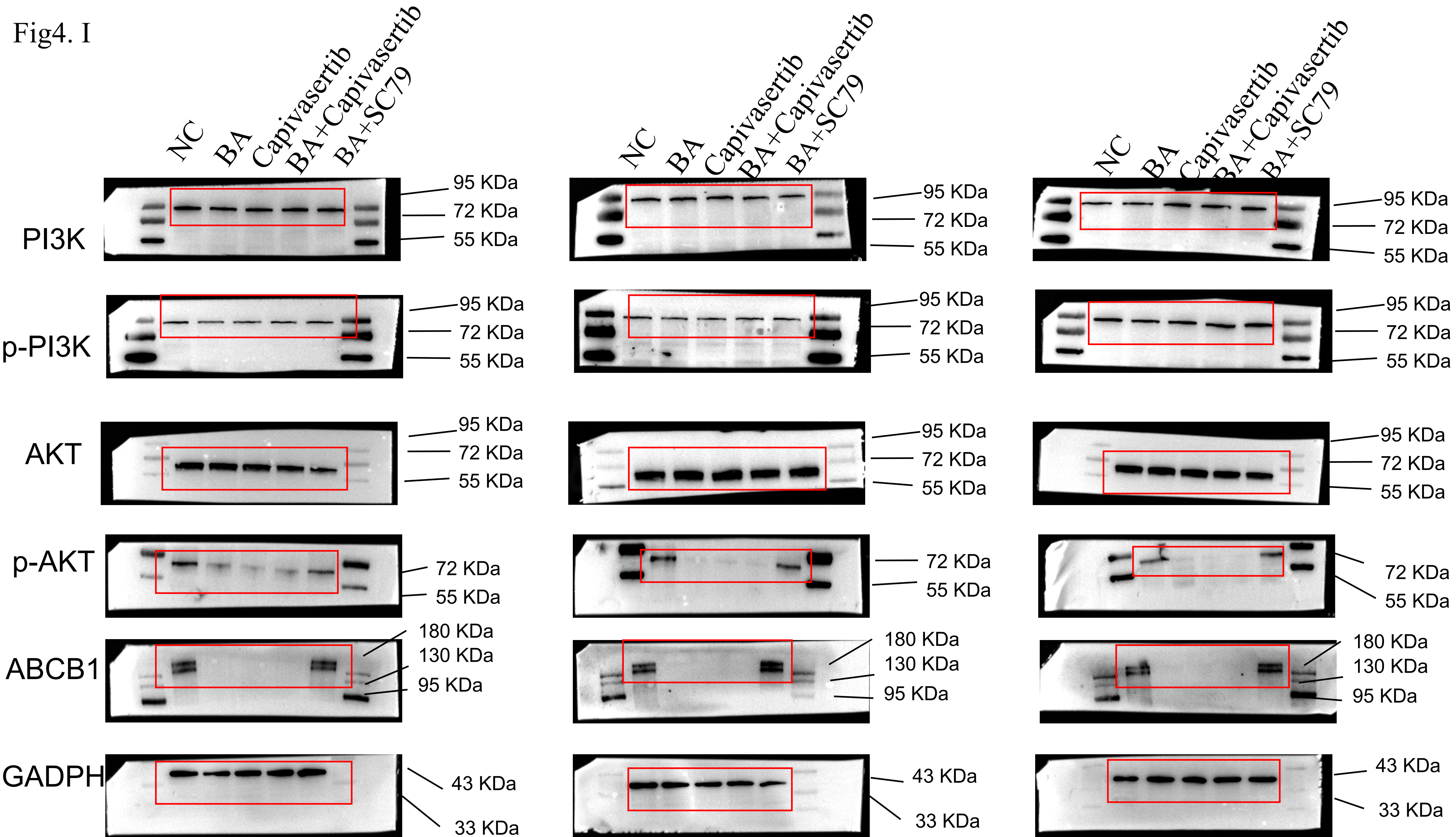
NC
BA
Capivasertib
BA+Capivasertib
BA+SC79
PI3K
p-PI3K
AKT
p-AKT
ABCB1
GADPH
180 KDa
130 KDa
95 KDa
72 KDa
55 KDa
43 KDa
33 KDa

Fig4. J

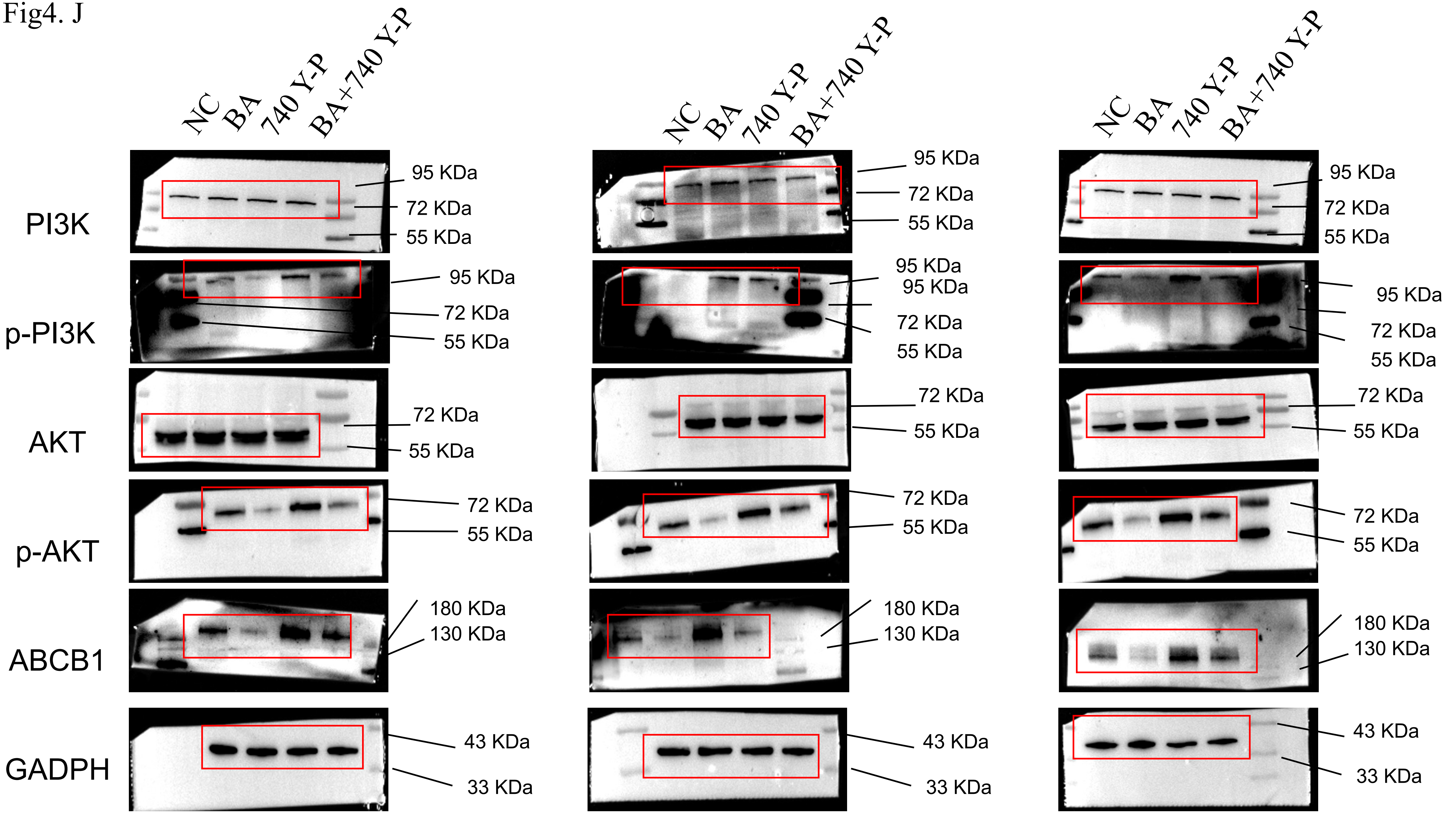

NC
BA
740 Y-P
BA+740 Y-P
PI3K
p-PI3K
AKT
p-AKT
ABCB1
GADPH
95 KDa
72 KDa
55 KDa
180 KDa
130 KDa
43 KDa
33 KDa

Fig4.K

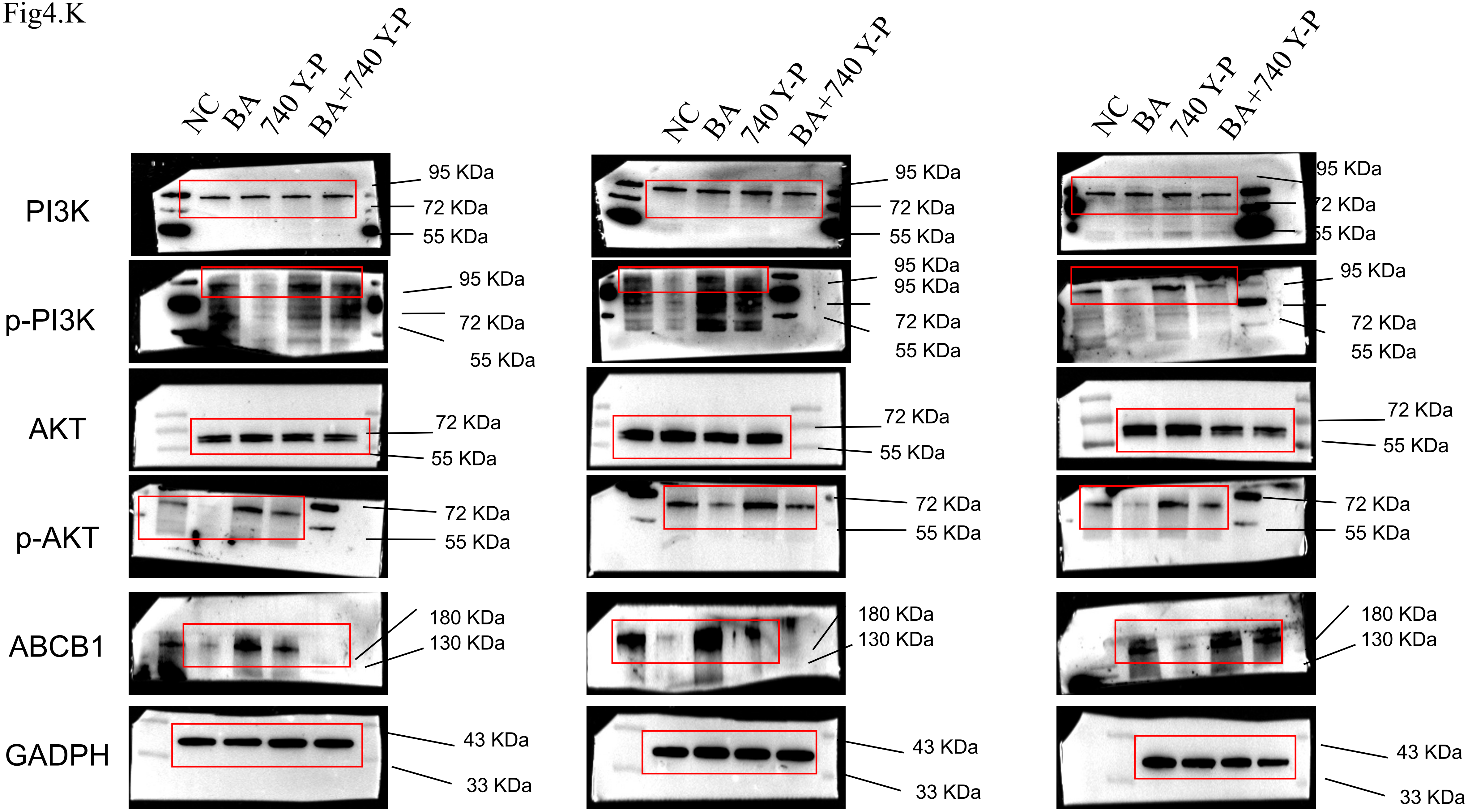
NC
BA
740 Y-P
BA+740 Y-P
PI3K
p-PI3K
AKT
p-AKT
ABCB1
GADPH
95 KDa
72 KDa
55 KDa
180 KDa
130 KDa
43 KDa
33 KDa

Fig4. L

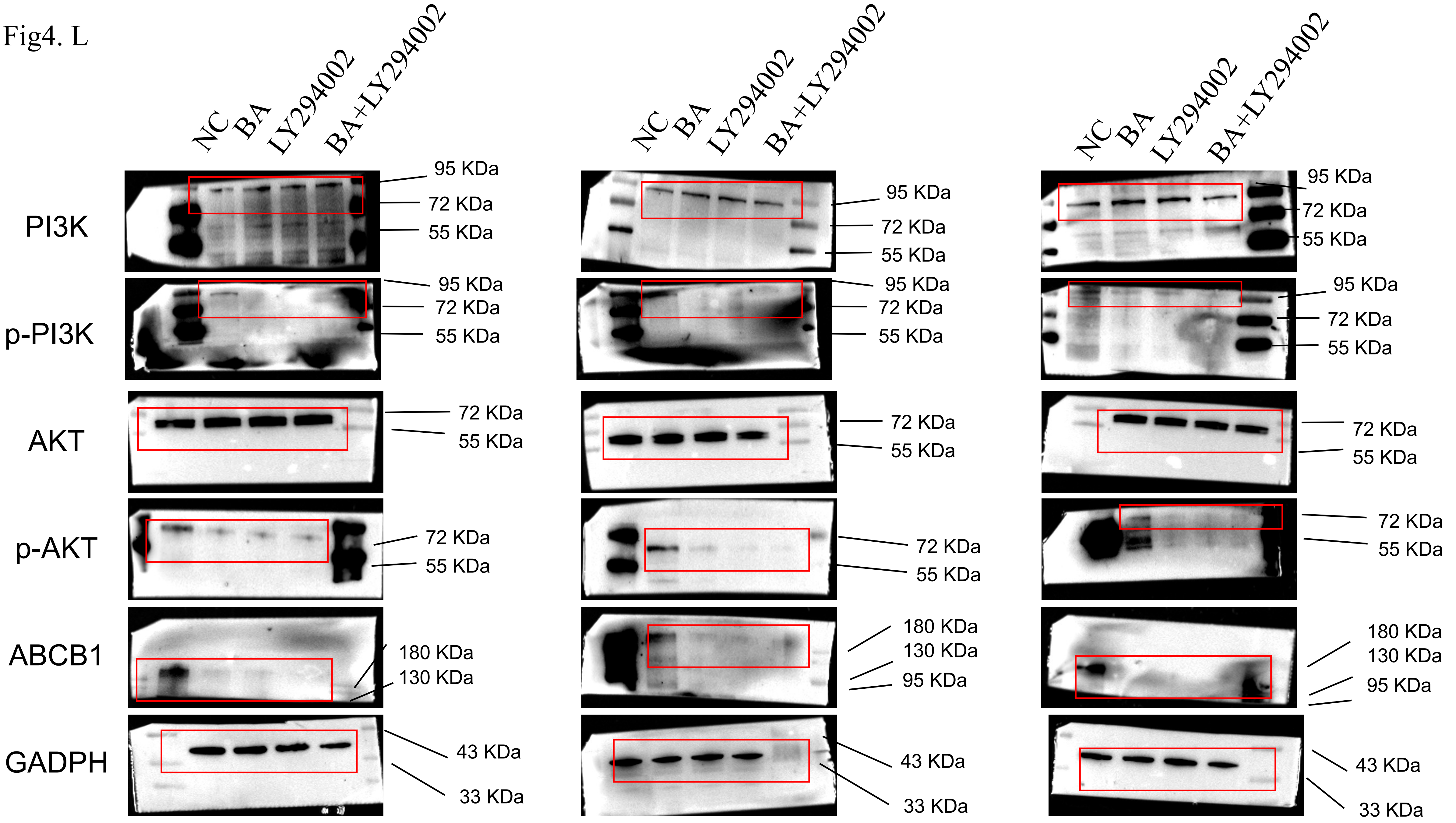
NC
BA
LY294002
BA+LY294002
PI3K
p-PI3K
AKT
p-AKT
ABCB1
GADPH
180 KDa
130 KDa
95 KDa
72 KDa
55 KDa
43 KDa
33 KDa

Fig4. M

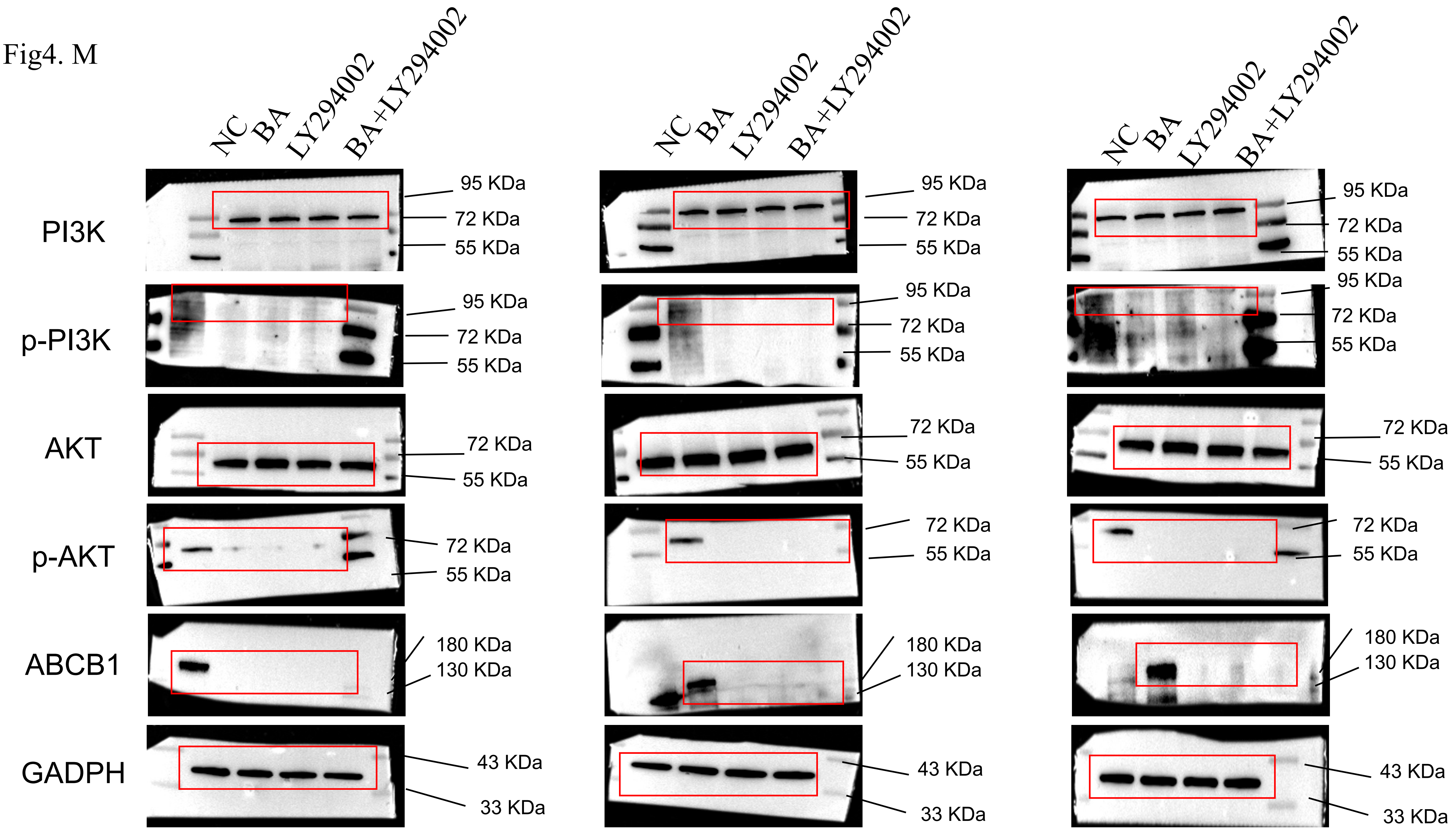
NC
BA
LY294002
BA+LY294002
PI3K
p-PI3K
AKT
p-AKT
ABCB1
GADPH
95 KDa
72 KDa
55 KDa
43 KDa
33 KDa
180 KDa
130 KDa

FigS1 I

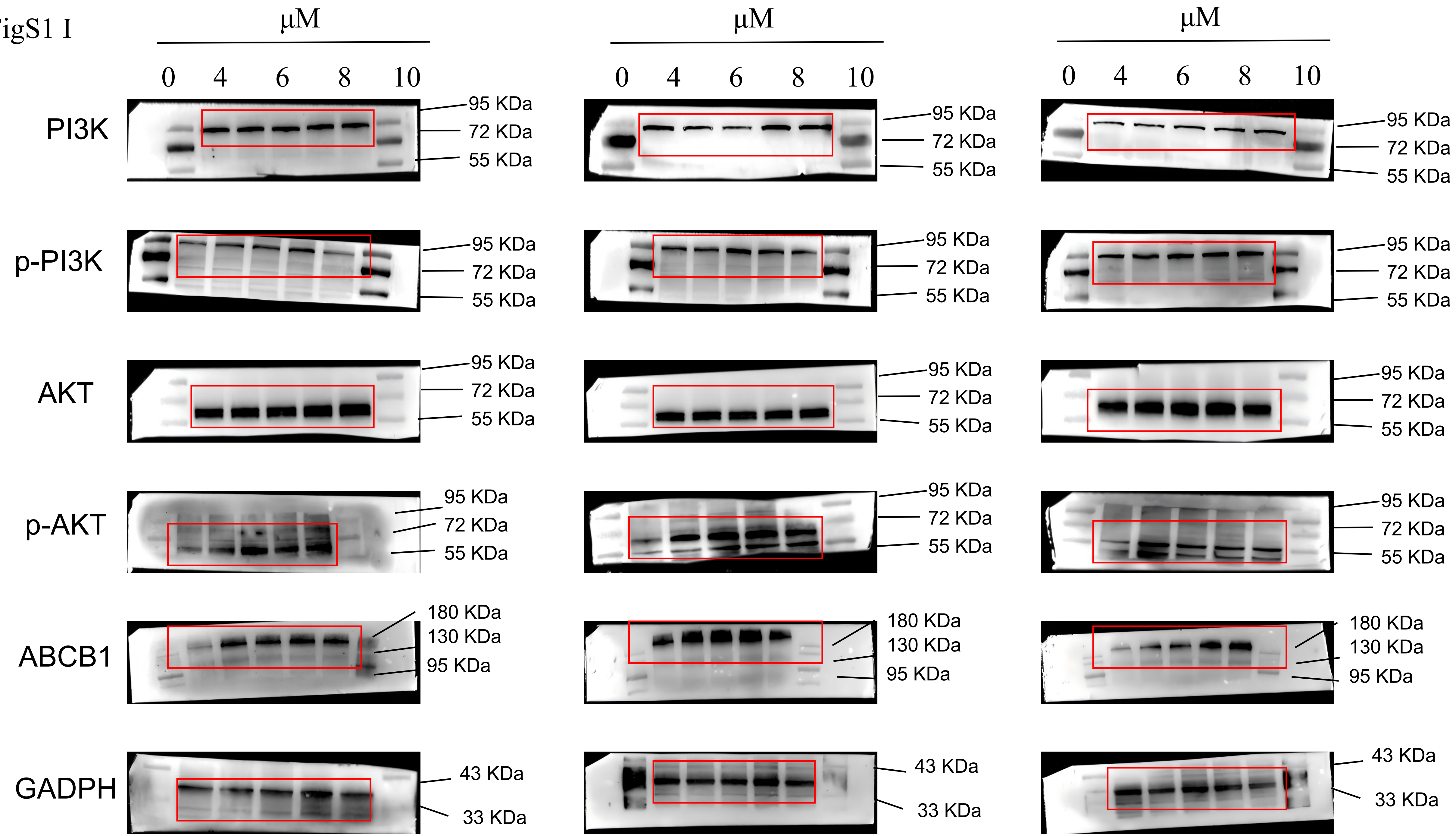
μM
0
4
6
8
10
PI3K
p-PI3K
AKT
p-AKT
ABCB1
GADPH
180 KDa
130 KDa
95 KDa
72 KDa
55 KDa
43 KDa
33 KDa

FigS1 J

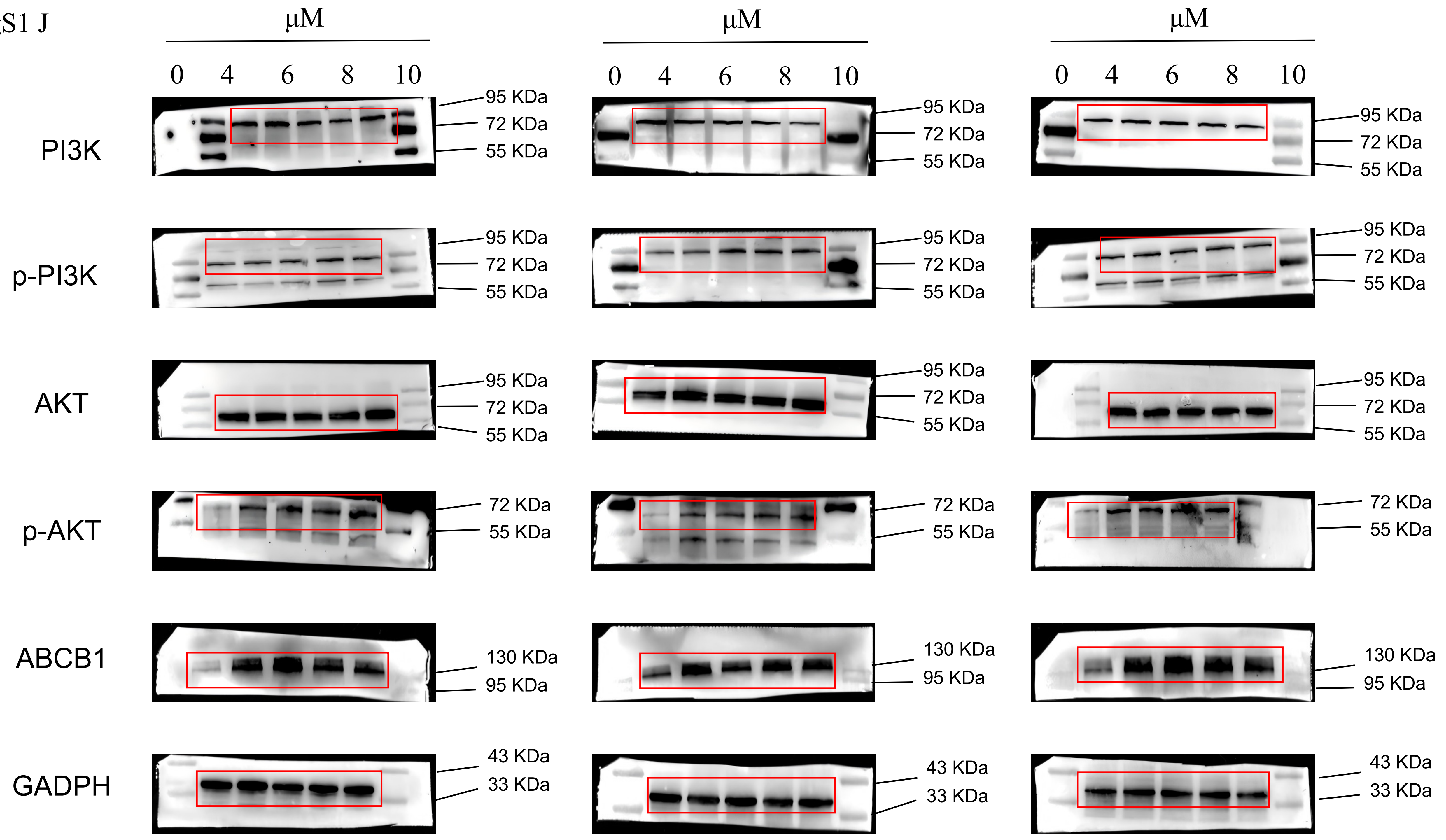
μM
0
4
6
8
10
PI3K
p-PI3K
AKT
p-AKT
ABCB1
GADPH
95 KDa
72 KDa
55 KDa
130 KDa
43 KDa
33 KDa

FigS1 K

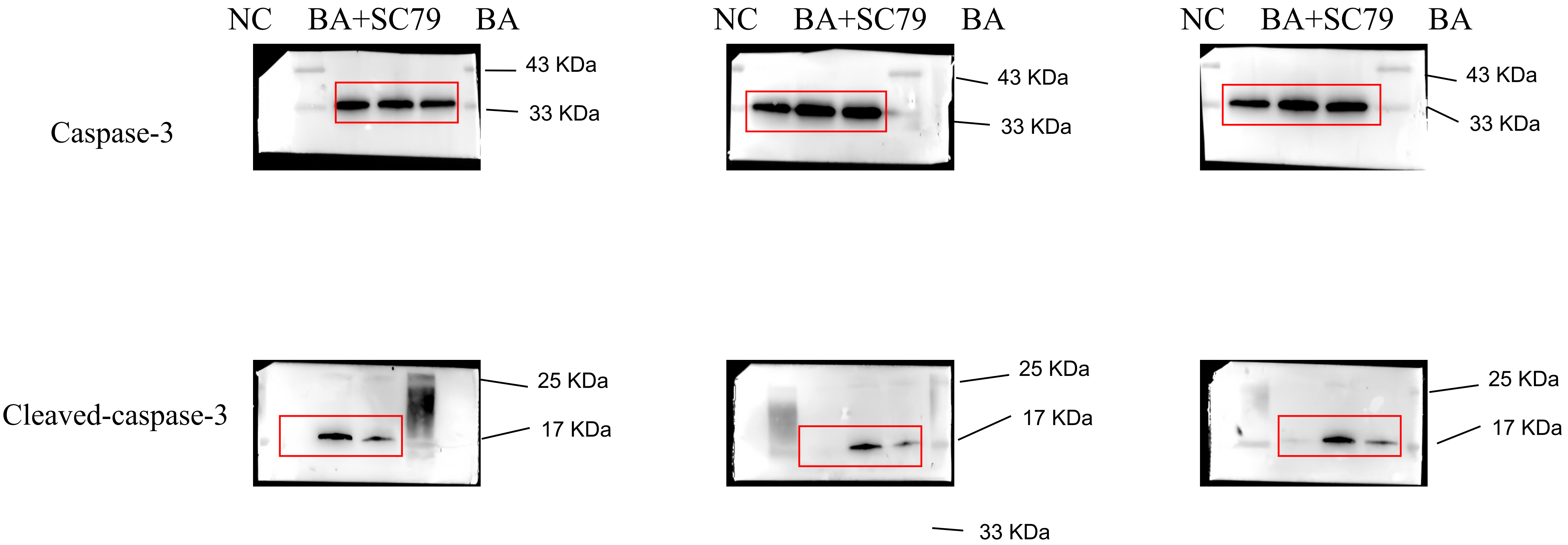
NC
BA+SC79
BA
NC
BA+SC79
BA
NC
BA+SC79
BA
Caspase-3
43 KDa
33 KDa
43 KDa
33 KDa
43 KDa
33 KDa
Cleaved-caspase-3
25 KDa
17 KDa
25 KDa
17 KDa
25 KDa
17 KDa
33 KDa

FigS1. L

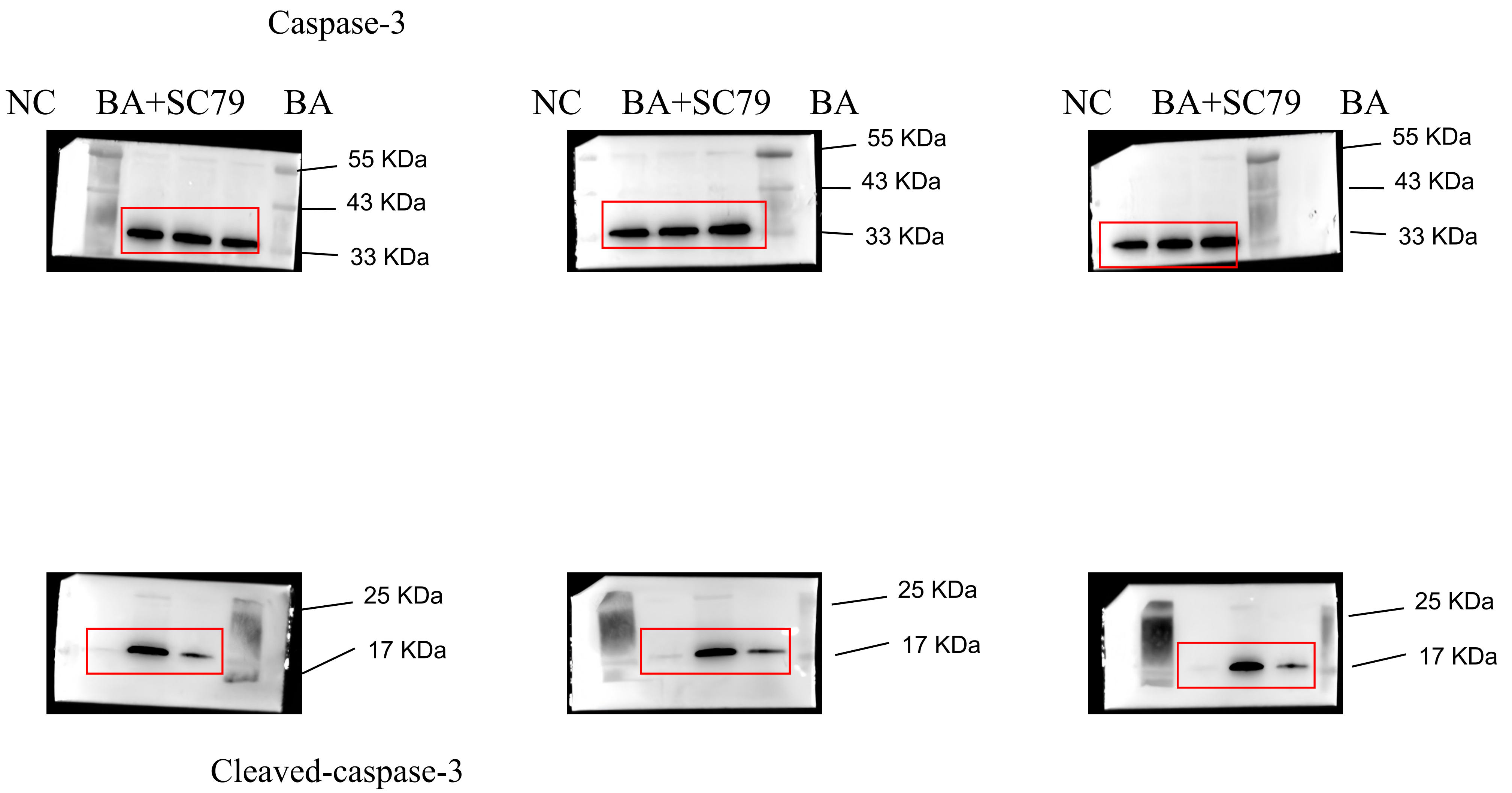
Caspase-3
NC BA+SC79 BA
NC BA+SC79 BA
NC BA+SC79 BA
55 KDa
43 KDa
33 KDa
55 KDa
43 KDa
33 KDa
55 KDa
43 KDa
33 KDa
25 KDa
17 KDa
25 KDa
17 KDa
25 KDa
17 KDa
Cleaved-caspase-3
